# Identification and Dynamic Regulation of microRNAs Involved in Salt Stress Responses in Functional Soybean Nodules by High-Throughput Sequencing

**DOI:** 10.3390/ijms14022717

**Published:** 2013-01-28

**Authors:** Zhanghui Dong, Lei Shi, Yanwei Wang, Liang Chen, Zhaoming Cai, Youning Wang, Jingbo Jin, Xia Li

**Affiliations:** 1 Key State Laboratory of Plant Cell & Chromosome Engineering, Center of Agricultural Resources Research, Institute of Genetics and Developmental Biology, Chinese Academy of Sciences, Shijiazhuang 050021, Hebei, China; E-Mails: dongzhanghui2005@163.com (Z.D.); shilei711@126.com (L.S.); cl007z@yahoo.com.cn (L.C.); caizhaoming-2000@163.com (Z.C.); youningwang@163.com (Y.W.); 2 Graduate School of Chinese Academy of Sciences, 19A Yuquanlu Shijingshanqu, Beijing 100049, China; 3 Shijiazhuang Academy of Agriculture and Forestry Sciences, Shijiazhuang 050041, Hebei, China; 4 National Engineering Laboratory for Tree Breeding, Key Laboratory of Genetics and Breeding in Forest Trees and Ornamental Plants, College of Biological Sciences and Biotechnology, Beijing Forestry University, Beijing 100083, China; E-Mail: wangyanw321@tom.com; 5 Key Laboratory of Plant Molecular Physiology, Institute of Botany, Chinese Academy of Sciences; Beijing 100093, China; E-Mail: jinjb@ibcas.ac.cn

**Keywords:** functional nodules, miRNA, nitrogen fixation, salt stress, soybean

## Abstract

Both symbiosis between legumes and rhizobia and nitrogen fixation in functional nodules are dramatically affected by salt stress. Better understanding of the molecular mechanisms that regulate the salt tolerance of functional nodules is essential for genetic improvement of nitrogen fixation efficiency. microRNAs (miRNAs) have been implicated in stress responses in many plants and in symbiotic nitrogen fixation (SNF) in soybean. However, the dynamic regulation of miRNAs in functioning nodules during salt stress response remains unknown. We performed deep sequencing of miRNAs to understand the miRNA expression profile in normal or salt stressed-soybean mature nodules. We identified 110 known miRNAs belonging to 61 miRNA families and 128 novel miRNAs belonging to 64 miRNA families. Among them, 104 miRNAs were dramatically differentially expressed (>2-fold or detected only in one library) during salt stress. qRT-PCR analysis of eight miRNAs confirmed that these miRNAs were dynamically regulated in response to salt stress in functional soybean nodules. These data significantly increase the number of miRNAs known to be expressed in soybean nodules, and revealed for the first time a dynamic regulation of miRNAs during salt stress in functional nodules. The findings suggest great potential for miRNAs in functional soybean nodules during salt stress.

## 1. Introduction

Functional root nodules are specialized organs in root systems where symbiotic nitrogen-fixation takes place in legumes. These nitrogen-fixing nodules are composed of symbiosomes, which consist of a plant-originated peribacteriod membrane and nitrogen-fixing bacteriods [1,2]. The bacteriods in the functional nodules fix atmospheric nitrogen into ammonia that can be absorbed and assimilated rapidly by the plant cells in return for carbon energy from the host plants. The mutualistic symbiotic relationship established between legumes and nitrogen-fixing bacteria is a unique adaptive strategy for legume crops to cope with restricted availability of nitrogen in soil, limiting crop production during evolution. Effective symbiotic nitrogen fixation (SNF) within nodules is the most economical, cost efficient and eco-friendly way to provide sufficient nitrogen for leguminous plants and to ensure agricultural sustainability [3,4]. However, rates of SNF in legume plants are negatively affected by different environmental stresses. The massive decline in efficiency of SNF in soybeans and other legumes has been noticed over the last few decades [5,6]. Many factors, including environmental stresses and various chemicals used in agricultural practices affect the establishment of the symbiotic relationship between legume plants and their nitrogen-fixing rhizobia partners, as well as the nitrogen fixation processes themselves, resulting in reduced SNF and decline in crop yield [3,7]. Therefore, understanding how abiotic stresses affect SNF is crucial to maximizing symbiotic effectiveness for improvement of crop productivity and agricultural sustainability.

Salinity is one of the most severe environmental factors affecting plant growth and causing agricultural losses worldwide. Salt-affected area is up to 800 million ha, which accounts for about 6% of the world’s agricultural land [8]. High salinity imposes osmotic stress, ion toxicity and nutrient unbalance causing cell damage, growth inhibition and yield reduction of plants. As with the majority of cultivated crops, legume plants are very sensitive to high salinity and their growth and yield are greatly affected by salt stress, although their tolerance to salt stress varies among genotypes [9]. Previous studies have shown that the early steps in nodule initiation (colonization of Rhizobium on roots, root hair curling or deformation and nodule organogenesis) are very sensitive to salt [10–12]. Salt stress also induced a series of morphological and physiological alterations of nodules in legumes, such as decreased nodule respiration, accumulation of proline and soluble sugars, and reduced activity of nitrogenase catalyzing the fixation of atmospheric nitrogen into ammonia [13–16]. As a result, salt stress-induced great reduction in nitrogen fixation efficiency. However, the molecular mechanisms underlying salt tolerance of symbiosis and nitrogen fixation efficiency in legumes are poorly understood. In *Medicago spp.*, MtCPK3, an isoform of calcium-dependent protein kinases (CDPKs), is rapidly induced during nodulation under salt stress [17]. A recent study about miRNA using salt-stressed chickpea roots and nodules detected a differential organ-specific response to salt stress by examining global transcriptome changes using deepSuper SAGE [18]. Nevertheless, the precise mechanisms of MtCPK3- and salt-responsive genes-mediated stress response to salt during nodulation and SNF remain elusive.

MicroRNAs (miRNAs) are small (20–24 nucleotides) non-coding RNAs which bind to the CDS or untranslated regions (UTR) of target genes, resulting in reduced expression of the proteins. Reduction of the protein levels is mainly achieved by accelerating mRNA decay or repressing translation of the target mRNAs [19–21]. The spatial and temporal patterns of miRNA expression during various biological processes ranging from organ development to stress responses in different organisms demonstrate critical regulatory roles for miRNAs [22]. Being sessile, plants have to cope with a constantly changing environment to guarantee their survival and reproductive success. Currently, there are several evidences that make us strongly suggest that miRNA are involved in abiotic stress. In *Arabidopsis*, several genes in abscisic acid signaling (ABA) and stress response were found to be involved in miRNA biogenesis [23]. Genome-wide screens have identified miRNAs, such as miR393, miR394, miR396 and miR156 specifically responsive to salt stress in *Arabidopsis*, *Zea mays*, *Populus tremula*, rice and soybean [21,24–29]. Interestingly, some novel stress responsive miRNAs were found unique to rice [30]. These findings suggest that miRNAs play vital roles in plant adaptation to salt stress, and different plant species or organs of a single species may cope with salt stress using different miRNA-mediated regulatory strategies.

Several recent reports have revealed important regulatory roles for small RNAs in controlling nodulation and SNF in the legumes *Medicago trincatula* and soybean (*Glycine max*). Mtr-miR169 was found to regulate nodule development through regulating expression of transcription factor *MtHAP2-1* in *Medicago truncatula* [31]. In soybean, gma-miR168, gma-miR393 and gma-miR172 are induced during early interaction between soybean roots and *Bradyrhizobium japonicum* [32]. Overexpression of gma-miR482, gma-miR1512 and gma-miR1515 in roots significantly increases soybean nodule number without affecting root length, lateral roots or number of nodule primordia, suggesting critical roles of these miRNAs during nodule development [33]. Differential expression of miRNAs such as gma-miR167, gma-miR172, gma-miR399, gma-miR396 and gma-miR169c in soybean nodules, and mtr-miR2568, mtr-miR107 in *M. truncatula* were also identified [34–36]. Therefore, miRNAs are involved in various steps during symbiosis establishment and SNF. However, the dynamics of miRNA expression during symbiosis, particularly at the late stage of nodule development and the nitrogen-fixation stage, has so far not been investigated. It also remains unclear whether expression levels of any miRNAs are specifically regulated in salt-stressed nodules and could thereby control nitrogen fixation efficiency in response to salt stress. To address these issues, we applied Solexa high-throughput sequencing to both libraries from non-stressed and salt-stressed mature nodules. We report here the results of the first global miRNA screen identifying differences in miRNA expression between non-stressed and salt-stressed nodules. Our results revealed that miRNA-mediated genetic pathways play important roles during late nodule development and nitrogen fixation control.

## 2. Results and Discussion

### 2.1. Deep Sequencing of Small RNAs of Stressed and Non-Stressed Nodules

We have previously identified 32 miRNAs that belong to 11 miRNA families in a miRNA library from functional nodules harvested 28 d postinoculation with *Bradyrhizobium japonicum* using a cloning and sequencing approach [34]. In this study, we analyzed two small RNA libraries from non-stressed (NSN) and salt-stressed functional nodules (SSN) at the same stage using Solexa deep sequencing technology to enrich our understanding on miRNA-mediated regulatory mechanisms involving efficient control of nodule function and adaptation to salinity.

**Figure 1:**
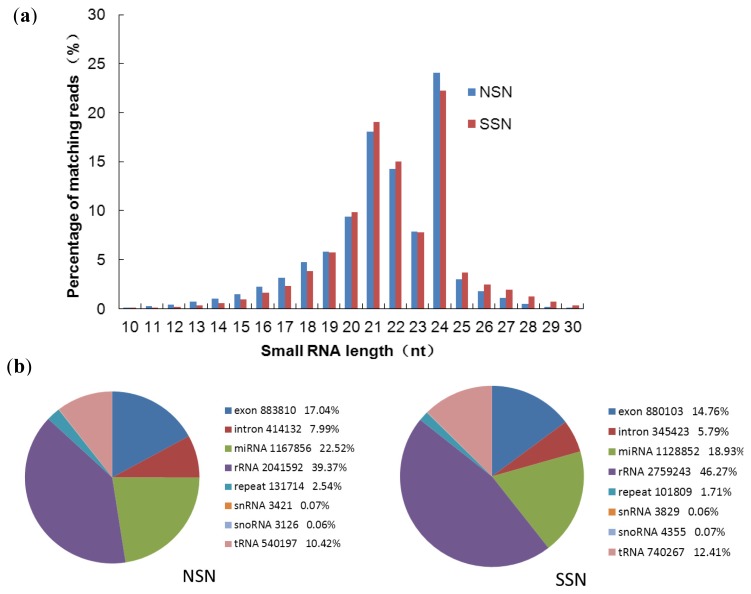
Characteristics of soybean mature nodule small RNAs. (**a**) Size distribution of small RNA raw reads from nonstressed (NSN)- and salt stressed (SSN) nodules. (**b**) Comparison and classification of small RNAs found in the NSN (**left**) and SSN (**right**) libraries. The mature nodules at 28 days after inoculation with USDA110 with and without 125 mM NaCl (6 h) treatment were harvested for small RNA extraction and the Solexa deep sequencing.

We obtained 23,252,859 and 23,794,887 sequencing raw reads from non-stressed and salt-stressed functional nodules respectively. The vast majority of the small RNA sequences from both libraries were 20–24 nt in length, and 24 nt RNAs were most abundant, followed in abundance by 21 nt, 22 nt, 20 nt and 23 nt long sequences (Figure 1a). The distribution pattern of small RNAs of both nonstressed and salt-stressed mature nodules in soybean is consistent with the previous results from other plants, as well as the typical size of miRNA from Dicer difestion products [37,38]. The number of the sequences between 18 and 30 nt in size were 12,303,456 and 12,608,512 for NSN and SSN respectively (Table S1). The remaining sequences were either of low quality (readings without reliable 3′ adaptor sequence) or smaller than 18 nt, and were excluded from further analysis. The 20–24 nt sequences from these two libraries were aligned to the global *Glycine max* genome, and a total of 7,607,757 (NSN) and 7,667,513 (SSN) sequences were found to match the genome perfectly. Finally we obtained a total of 3,589,765 and 2,832,484 sequences for non-stressed and stressed nodules after removing the sequences corresponding to known rRNAs, tRNAs, small nuclear RNAs and small nucleolar RNAs (Figure 1b and Table S1). Interestingly, we found that the abundance of small RNAs between 25 and 30 nt in NSN was lower than that of SSN (Figure 1a). When the data between the two libraries were compared, we observed that as with the perfectly matched sequences total, the number of the non-coding RNAs in the SSN library was higher than that from the NSN library. In particular, the non-coding sequence for rRNA in the SSN was 6.9% higher than that in the NSN (Figure 1b). In contrast, the number of sequences matched to coding RNAs in the SSN library was lower than that in the NSN (Figure 1b). As an important crop with high demand for nitrogen, SNF is essential for soybean yield and seed quality [39,40]. With the rapid development of new technology, genome wide sequencing technology allows detecting and profiling miRNAs at unprecedented sensitivity. Joshi *et al.* reported identification of miRNAs in four different soybean tissues including young nodules [41]. Their results greatly enriched the number of known miRNAs in nodulation and nodule organogenesis. However, they only focused on small RNAs distribution pattern in young nodules grown under normal conditions. Here, we reported for the first time the trend change in abundance of different sizes of small noncoding RNAs with or without salt treatment, and also the dynamics and identities of these small RNA changes in functional soybean nodules in response to salt stress. The dynamic changes in small RNAs may reflect regulatory functions of small miRNAs during nodule development and response to salt stress.

### 2.2. Identification and Conservation of Soybean Nodule miRNAs

**Figure 2:**
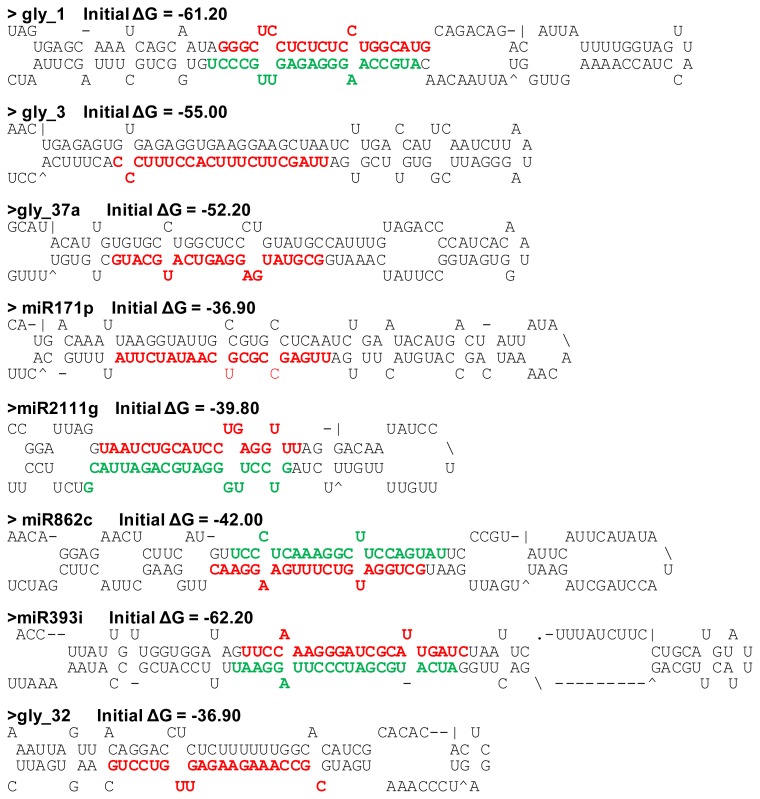
Predicted RNA hairpin structures of novel miRNA precursors. Precursor structures were predicted by Mfold online [43]. Mature miRNAs are highlighted in red and miRNA* in green.

**Figure 3:**
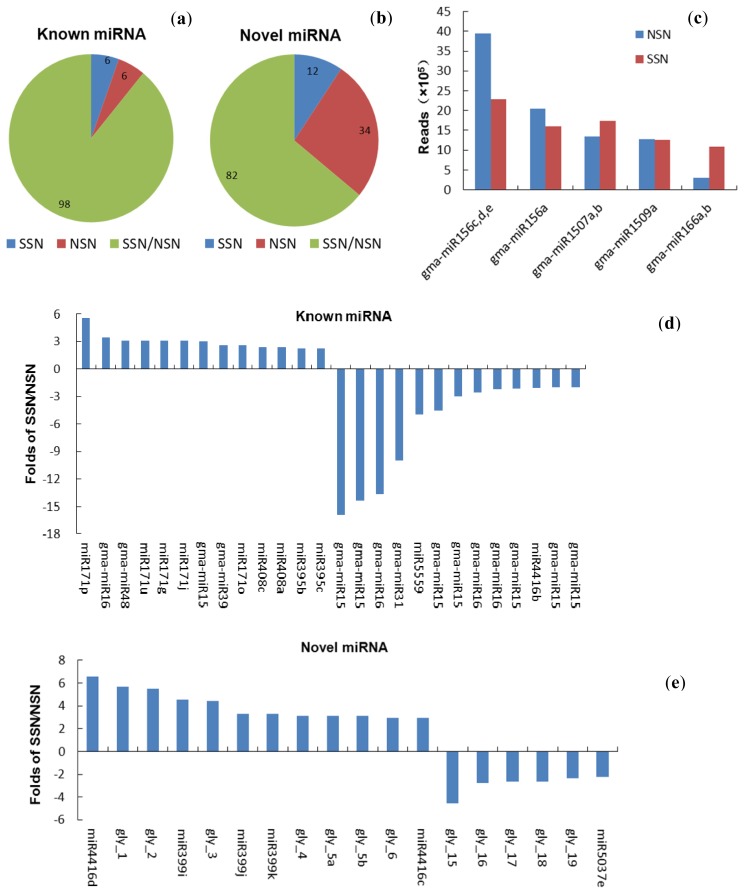
miRNAs in mature soybean nodules under normal conditions and salt stress. (**a**)(**b**) Diagram illustrating the relationship between the known (**a**) and novel (**b**) miRNAs identified in salt stressed- (SSN), nonstressed (NSN) or in both (SSN/NSN) libraries. (**c**) The known miRNAs highly expressed in NSN and SSN libraries. (**d**) The known miRNAs highly responsive to salt stress with a greater than 2-fold change over control. (**e**) Top regulated novel miRNAs with changes in expression levels greater than 2-fold by salt stress.

Next, we used de novo analyses to identify miRNAs as described in the materials and methods. Stem–loop hairpin structures of all novel miRNAs’ precursor are listed in Figure S1 and eight of these are presented in Figure 2. In total, we identified 220 and 194 miRNAs in the NSN and SSN respectively. To date, 555 miRNAs have been identified in soybean and registered in the miRNA database [42]. To find which miRNAs we identified are known miRNAs, putative miRNAs were aligned with known miRNA in soybean. As a result, the seed sequences for 104 mature nodule miRNAs belonging to 58 families from the NSN library were known miRNAs, whereas 104 known miRNAs grouping into 55 families were found in the SSN library (Table S2). Thus, we identified 116 and 94 novel miRNAs in the NSN and SSN libraries respectively (Tables S3 and (S4). Interestingly, we found that both NSN and SSN shared nearly all the known miRNAs (Figure 3a). However, when we compared the novel miRNAs in the two libraries, we found that 82 novel miRNAs were detected in both libraries, whereas 34 and 12 of the remaining novel miRNAs were only found in either the NSN or the SSN library (Figure 3b). Clearly, more putative novel miRNAs were identified in the NSN library, although the number of the known miRNAs was same. The data suggested that expression of some novel miRNAs was regulated by salt stress. However, the average abundance for each novel miRNA in the SSN library was much higher, indicating activation of miRNA by salt stress.

Recently, many miRNAs derived from shoot apical meristem, leaves, roots, flowers and seeds in soybean seedlings have been reported [38,41,44,45]. All miRNAs identified in our previous study [35] and some miRNAs such as miR156, miR159, miR1508, miR1509, miR1511-15, miR1518-23 *etc.*, found in *Bradyrhizobia* inoculated soybean roots [32], and gma-miRNAs, such as miR172, miR169, miR1511, miR156, miR4357, miR4416 *etc.*, from different tissues of soybean [41] were retrieved in this study. When comparing the top 10 highly abundant miRNAs identified from our experiment with those from other tissues of soybean which are available, we found that the top 10 highly expressed miRNAs in different tissues are dramatically distinct. For example, very few (1 to 2 miRNAs) in the top 10 most abundant miRNAs were shared by mature nodules and shoot apical meristem or nodules and young leaves in soybean, though almost all of them were detected in all tissues (Table S5). However, there were 5 miRNAs (gma-miR159a, gma-miR482, gma-miR168, gma-miR1511 and gma-miR2109), 50% of the top 10 miRNAs, identical in shoot apical meristem and young leaves of soybean (Table S5), which are functionally closely related in aboveground. It has been shown that miR159 acts as a molecular switch only permitting the expression of GAMYB-like genes which participate in GA-induced pathways in anthers and seeds [46]. High levels of miR159a expression may restrict activation of MYB transcription factor in active growing vegetative tissues. These results support the claim that each tissue has a specific pattern of miRNA expression, and functionally related/similar tissues or organs would share higher levels of similarity in miRNA expression. Therefore, we conclude that the miRNA expression pattern detected in this study may represent a unique pattern associated with the function of nitrogen fixation in mature soybean nodules.

To investigate the evolutionary conservation relationship of the novel miRNAs, the sequence similarity of 128 novel miRNAs representing 64 families were compared with other known miRNA in the miRBase database of *Glycine max*, *Medicago truncatula*, *Lotus japonicus*, *Phaseolus vulgaris*, *Arabidopsis thaliana*, *Oryza sativa*, *Vitis vinifera*, *Populus trichocarpa* and *Zea mays*. BLASTn search with an E-value cutoff of 10 was employed to search for the miRNAs sequence against the central miRNA Registry Database [42]. We found that 66 miRNAs representing 27 known miRNA families had identifiable locus in these plant species, 12 miRNAs are conserved in legumes, and strikingly 10 miRNAs (miR1513d, miR1520s, miR4357b,c, miR4416b, miR4416c, miR5037e, miR862c, miR1507d, miR4405b, miR862d) were only found in soybean (Table S6). Our data also suggests that while there is high conservation of some miRNAs in functional nodules among closely related legumes, there is also significant miRNA evolution and diversity.

### 2.3. Expression of Known miRNAs in Nodules under Salt Stress

Several studies have reported that miRNAs are dynamically expressed in response to salt stress in various plants [21,26,47]. To investigate whether miRNAs are involved in salt tolerance in mature nodules and which miRNA or miRNA families play critical roles in mature nodules in this process, we compared the expression profile of miRNAs with and without salt treatment. Of 110 known miRNAs found in NSN and SSN libraries, 47 and 46 miRNAs respectively revealed increased or decreased expression in the SSN compared with that in the NSN (Table S2). 14 miRNAs were up-regulated at least 2-fold in the SSN, while 14 miRNAs were down-regulated more than 2-fold in the SSN (Figure 3d or Table S2). Among them, gma-miR159c, gma-miR159b, gma-miR169c and gma-miR319a,b were most downregulated (>10-fold) by salt stress.

When expression of different categories of miRNAs in abundance were analyzed in stressed nodules, we found that of the 14 least expressed miRNAs, 9 exhibited altered expression patterns (4 upregulated and 5 downregulated), whereas the remaining 5 did not show significant difference in abundance (Table S2). In the second least abundant miRNA categories, nearly one third miRNAs were up- or downregulated dramatically. The top 9 most highly expressed miRNAs (reads >100,000) also showed differential expression between NSN and SSN. Of them, expression of gma-miR1509a remained unchanged, and gma-miR166a,b showed a greater than 3-fold change in expression level (Figure 3c). Hence, the majority of the known miRNAs are differentially expressed in response to salt stress.

### 2.4. Expression of Novel miRNAs in the Salt Stressed Nodules

Firstly, we compared the abundance of novel miRNAs (nov-miRs) and found 82 miRNA expressed in both NSN and SSN. Of 82 miRNAs, a majority of nov-miRs were responsive to salt stress, and 12 and 6 were up- and downregulated over 2-fold by salt stress respectively (Table S3 or Figure 3e). Three miRNAs, miR4416d, gly_1 and gly_2 showed greater than 5-fold increases in abundance upon salt stress in mature soybean nodules, no one nov-miR had a more than 5-fold decline in abundance. Interestingly, none of them were the most abundant miRNAs.

To identify nov-miRs specifically responsive to salt stress, we thoroughly compared the nov-miRs between NSN and SSN. Notably, 34 nov-miRs were only detected in NSN, and became undetectable after mature soybean nodules were treated with salt for 6 h (Table S4). All of these nov-miRs are not high abundant miRNAs, and only 11 of them were over 50 reads (Table S4). The read frequency for gly_37a,b were highest in this group. Moreover, 12 nov-miRs were only found in SSN (Table S4). Of them, 9 were over 50 reads and miR1513e had highest abundance with read frequency 475, followed by miR167k with read frequency 343 (Table S4). It is likely that 34 nov-miRs were completely repressed, whereas 12 nov-miRs were activated when the mature soybean nodules were subjected to salt stress. Therefore, these nov-miRs potentially mediate the specific response to salt stress in mature soybean nodules.

### 2.5. Experimental Validation of the Salt-Responsive Expression of miRNAs

**Figure 4:**
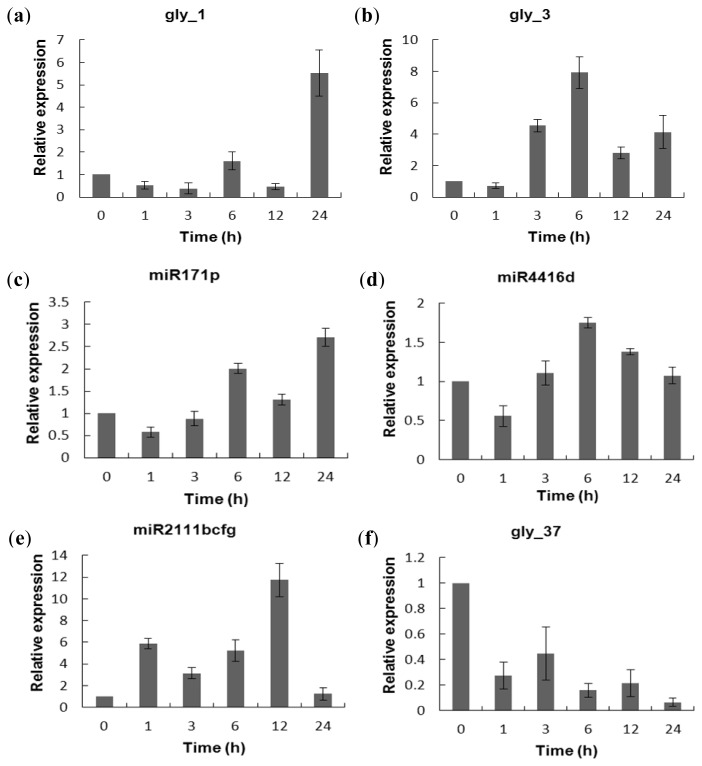

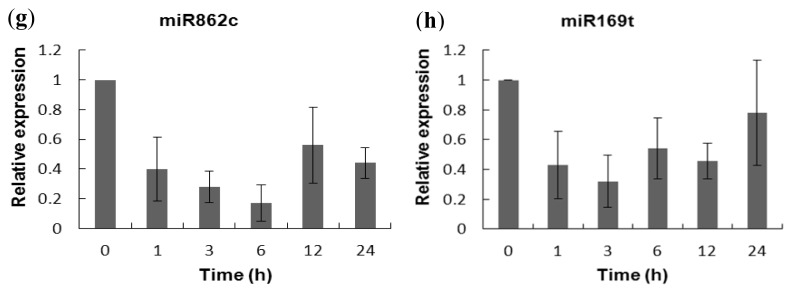
qRT-PCR analysis of the miRNAs in response to salt stress in functional nodules. Small RNAs were isolated from functional soybean nodules 28 days after *B. japonicum* strain USDA110 inoculation treated with 125 mM NaCl for 0, 1, 3, 6, 12, 24 h. (**a**) to (**d**) The miRNAs induced by salt stress; (**e**)(**f**) The ovel miRNAs only detected in SSN library in sequencing data; (**g**)(**h**) The miRNAs were only detected in NSN library in sequencing data. Error bars indicate standard devation. 5.8srRNA was used as internal control and the normalized miRNA levels at 0 h were set to 1. Each experiment consisted of three biological replicates.

To validate the expression patterns of the miRNAs identified in response to salt stress, we performed the qRT-PCR for some miRNAs using the mature nodules treated with or without salt stress at the specified time points. As shown in Figure 4, expression of the selected miRNAs highly induced by salt (gly_1, gly_3, miR171p and miR4416d) showed similar trends as previously detected in the Solexa sequencing data, although the exact levels or peaks of expression for each miRNA were somewhat variable. Of them, gly_1 and gly_3 were strongly induced by salt stress, but miR171p and miR4416d showed less of an increase (Figure 4a–d). Time course analysis of miRNA expression over 24 h in salt-treated nodules also identified miRNAs with differential expression. For example, expression of gly_3 peaked at 6 h after salt treatment, but restored to its original level by prolonged salt treatment (Figure 4b). The results demonstrate that our sequencing data are reliable and quantitative and miRNAs differentially regulated salt response in mature soybean nodules.

We next checked the expression of several miRNAs by qRT-PCR, which were detected only in SSN or NSN in the Solexa data. For instance, miR2111b,c,f,g which was only detected in SSN, showed very high levels of induction by salt in qRT-PCR analysis (Figure 4e), but expression of this miRNAs was also detected in the nodules grown under normal conditions, though their expression levels were quite low. By contrast, gly_37, miR862c and miR169t detected only in the NSN library displayed the highest levels of expression under control conditions (Figure 4f–h). We analyzed the miRNAs’ qRT-PCR experiment by DPS and the result showed in Table S7.

Until now, few works on identification of soybean miRNAs in response to abiotic stresses have been reported. However, to our knowledge, this is the first report on miRNA identification in soybean functional nodules obtained from salt-treated plants by deep sequencing. By analyzing the enrichment and depletion of miRNAs derived from salt-treated and non-treated nodules, we revealed a specific expression pattern of miRNAs unique to soybean mature nodules, and demonstrate differential expression of miRNAs in response to salt stress. Expression validation of the novel miRNAs responsive to salt stress in nitrogen fixing nodules confirmed that miRNAs are critical regulators in nodule adaptation to high salinity during nitrogen fixation process. Further analysis of their targets and functions of target genes during this process will provide novel insights into the molecular mechanisms of salt response in functional nodules of soybean.

### 2.6. Gma-miRNAs Target Prediction

miRNAs regulate various biological processes through negatively regulating their target genes [48]. To further understand the roles of the miRNAs, we predicted putative target genes of the identified miRNAs in our study. In total, 770 target candidates for 224 miRNAs were identified using miRNA target prediction algorithms (Table S8). Among them, there were 411 and 368 target genes for the known and novel miRNAs respectively. The number of the predicted targets varies extensively among miRNAs from 0 to 18. The majority of miRNAs had multiple targets (2 to 10 targets), 17 miRNAs had only one target candidate, and 20 miRNAs had more than 10 target genes. The majority of miRNAs targeted functionally diverse classes of genes; among them, gly_11 is likely to regulate 12 functionally different target genes. We found that 23 miRNAs targeted the genes encoding different members of serine-threonine protein kinase family, but no single gene was targeted by multiple miRNAs. The alignments between some identified novel miRNAs and the predicted targets were shown in Table 1. (**g**) (**h**) We can see complete or nearly complete reverse complementary matches between the miRNA and their targets.

The previous microarray analysis of young nodules in soybean suggests that nodulation and functions of symbiosis are regulated by complex regulatory networks at multiple levels [49]. Among 770 predicated targets, there were 79 transcription factors (TFs), such as B-box zinc finger, homeodomain protein, AP2 TFs etc. In particular, among 26 putative targets for the top 9 highly expressed miRNAs, 14 targets were TFs, including 1 zinc finger protein and 11 SBP TFs. In addition, the predicated targets included genes in protein biosynthesis/degradation (*i.e.*, Ca^2+^/calmodulin-dependent protein kinase, F-box proteins, ubiquitin-conjugating enzymes, proteases *etc.*), suggesting that nodule functions and stress response are regulated at multiple levels. Importantly, we found 2 putative targets annotated as an inorganic pyrophosphatase/nucleosome remodeling factor by a novel miRNA gly_20 and a symovial sarcoma-associated SS18 protein which interacts with SWI/SNF protein in nucleus for miR168, suggesting the chromatin remodeling plays a critical role in functioning of SNF. Strikingly, a hemoglobinase family protein involving hemoglobin catabolism was predicated as a putative target for a novel miRNA, gly_32, suggesting that gly_32 may directly regulate the function of nitrogen fixation in mature soybean nodules. Furthermore, we found that many putative target genes might play important roles in various biological processes, such as cell cycle (Translin and translin associated protein, CDC2-related kinase), cellular skeleton (tubulin-specific chaperone, tubulin targeted by a miR156 variant), lipid/carbohydrate metabolism and ion/anion/sugar/amino transport. Twenty genes targeted by seven miRNAs are likely involved in auxin synthesis, auxin response and auxin regulated morphorgenesis. Many genes annotated as TFs, phosphotases, and kinases modulate plant responses to abiotic and biotic stresses. Moreover, several targets act in antioxidant defense, such as peroxidase, glutaredoxin, NADH dehydrogenase, *etc.*). Notably, glutamine amidotransferase class-I, a key enzyme involving glutamate synthesis from glutamine was also identified as the target of miR1511. Expression in high levels of gma-miR1511 in functionally very different tissues suggests that conversion of glutamine to glutamate occurs in all tissues and that miR1511 is essential for regulation of nitrogen metabolism in soybean. This makes sense because nitrogen demands are higher during soybean growth and development than in non-legume crops [50].

The second goal of this study is to reveal the expression profile of mature nodules under salt stress and to find the miRNAs that regulate nodule response to salinity. Our comparative analysis revealed that up to 43.3% of the identified miRNAs were dramatically differentially expressed in salt-stressed and non-stressed mature nodules. Forty six miRNAs are down- or up-regulated over 2-fold by salt (Tables S2 and S3), and 58 were only detected in nodules grown under either normal or stress conditions (Tables S2 and S4). Bioinformatic prediction of the potential targets reveals that many of them have been reported as associated with stress response [21,27,51]. Among them, some genes serve as TFs regulating abiotic stress responses through downstream genes and networks [52], some are important components in the stress signaling pathway, such as PP2C family and serine/threonine kinase. Many genes which are directly involved in transcription, post-transcription, protein biosynthesis, protein modification and proteosome-mediated protein degradation were targeted by stress responsive miRNAs. In addition, putative target genes include various transporters (K^+^ potassium transporter, manganese transporter, sulfate transporter, inorganic phosphate transporter) which have been implicated to play roles in ion and osmotic homeostasis [53,54]. These encouraging and interesting observations have linked these miRNAs to multiple components in the stress signaling pathways. Because miRNAs are considered as managers in gene regulatory networks, they may provide quantitative regulation of genes to optimize nodule cells’ response to salt stress.

In addition, the majority of the miRNAs highly expressed in nodules were also responsive to salt stress (Table S2 or Figure 3c). Among them, gma-miR166a,b showed the highest induction by salt among all the differentially expressed known-miRNAs. Gma-miR159c, gma-miR159b, gma-miR169c, gma-miR319a,b and gma-miR1517 exhibited a more significant downregulation between stressed- and non-stressed nodules (Table S2). These miRNAs and their targets such as MYB TFs and NFY-A have previously been linked to abiotic stress and ABA signaling [55,56]. Further analysis also revealed the association of several novel miRNAs with nodule response to salt stress. Gma-miR4416d, gly_1 and gly_15 and gma-miR5559 are respectively the most prominently upregulated and downregulated by salt stress (Table S3). Their predicted targets zinc finger TFs, GRAS, serine-threonine protein kinase, and calmodulin-dependent protein kinases have been linked to plant adaptation to abiotic stress in *Arabidopsis* [57–59]. Further characterization of these miRNAs and their targets in modulating nodule response to salinity is needed. Moreover, novel miRNA gly_32 was detected only in salt-stressed nodules (Table S4) and one target of gly_32 is a hemoglobinase family member which catalyzes the degradation of hemoglobin, which is essential in functional symbiosis between legumes and rhizobia [60]. In particular, hemoglobin was previously shown to be an adaptive outcome in legumes, and to play a significant role when nodules are exposed to adverse environmental conditions [61]. Therefore, gly_32 may inhibit activity of hemoglobinase in mature soybean nodules under salt stress, thus maintaining both high levels of hemoglobin in stressed nodules and the subsequent functioning symbiosis and nitrogen fixation activity. This is consistent with the evolutionary role of hemoglobin in adaptation to adverse environments. Further studies are needed to better elucidate causal relationships between gly_32 and hemoglobin catabolism as well as between gly_32 and nodule adaptation to high salinity.

**Figure 5:**
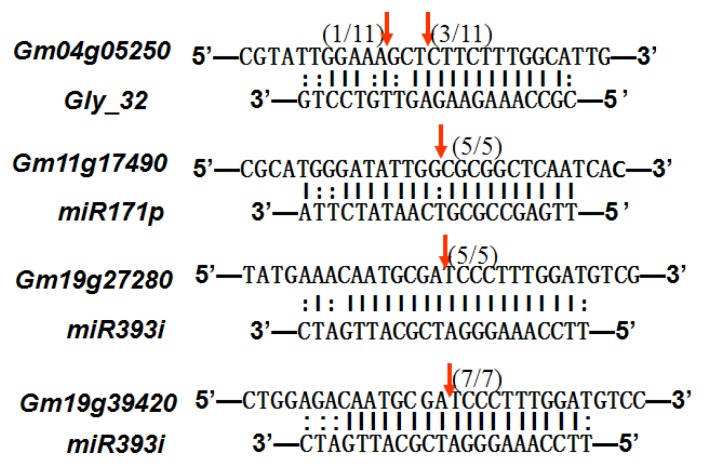
Cleavage sites validation of selected miRNA targets by 5′-RACE analysis. Accurate complementary bases between targets and miRNAs are shown connected by solid lines. The red arrows indicate the side of cleavage. The number is the frequency of accurate clones when validating the cleavage sites of target mRNA.

To experimentally validate the cleavage sites of the putative targets, we performed a 5′-RACE reaction for some putative targets. Figure 5 demonstrated that, *Glyma04g05250* was a target for gly_32*, Gm11g17490* was a target for miR171p and *Gm19g27280* and *Gm19g39420* for miR393i. The sequencing result showed that gly_32 cut its target between 14th and 15th or 11th and 12th nt, miR171p cut its target between 10th and 11th nt and miR393i cut its targets between 11th and 12th nt of the miRNAs’ 5′ end. The results suggest that miRNAs gly_32, miR171p and miR393i may modulate nodule functions through binding to their target mRNA and negatively regulating their expression. *Gm19g27280* and *Gm19g39420* code AFB2 and TIR1 like protein respectively, which indicate that auxin perception and signaling pathway may play a role in nodule function and stress response to salt stress.

## 3. Experimental Section

### 3.1. Plant Growth Conditions, Inoculation with *B. japonicum* and Salt Treatment

Seeds (*Glycine max* cv. *Houzimao*) were surface-sterilzied with 70% ethanol for 1 min, then washed 6 times with sterile water, and germinated in filter papers soaked with distilled water at 28 °C for 4 days in the dark. *B. japonicum* USDA110 was grown in TY liquid medium (5 g Tryptone, 3 g yeast extract, and CaCl_2_·2H_2_O 0.7 g) at 28 °C for 4–5 days. Germinated seeds were inoculated with a suspension (10^8^ cells/mL) of USDA110 for 30 min, and then transferred to bottles containing sterilized vermiculite saturated with a low nutrient Hoagland’s solution (Ca(NO_3_)·4H_2_O 0.03 g, CaCl_2_·2H_2_O 0.1 g, KH_2_PO_4_ 0.1 g, Na_2_HPO_4_·12H_2_O 0.15 g, MgSO_4_·7H_2_O 0.12 g, ferric citrate 0.05 g/L) plus 1 mL micro-element (H_2_BO_3_ 2.86 mg, 1.81 mg MnSO_4_, 0.22 mg ZnSO_4_, 0.8 mg CuSO_4_, and 0.02 mg/L H_2_MO_4_), which is suitable for *B. Japonicum* infection. The inoculated seedlings were grown in a growth chamber at 27 °C under a 16 h/8 h (light/dark) photoperiod with light intensity of 10,000 lux. Non-inoculated seedlings grown under the same conditions and on sterilized vermiculite saturated with Hoagland’s solution were used as controls. Plant roots were then hydroponically treated with 125 mM NaCl at twenty-eight days after inoculation with *B. Japonicum* (28 dpi), and the nodules were harvested 6 h after treatment.

### 3.2. Small RNA Library Construction and Solexa Sequencing

For small RNA library construction and high-throughput sequencing, the plants with mature nodules 28 days after inoculation were treated with 125 mM NaCl for 6 h, and the stressed mature nodules were harvested and immediately frozen in liquid N_2_. The nodules without salt treatment were collected as a control. Small RNAs were isolated from the stressed (SSN) and non-stressed nodules (NSN) using a mirVana-miRNA Isolation kit (Ambion, Austin, TX, USA), and were subjected for sequencing using the Illumina-Solexa 1 Genetic Analyzer in the Beijing Genomics Institute, following the manufacturer’s protocols. The read abundances of miRNAs in the two libraries were normalized to one million (normalized expression = actual miRNA count/total count of clean reads × normalized one order of magnitude).

### 3.3. Data Analysis

Clean sequence were screened from raw sequence by removing contaminant sequence including adapters, the inster tag, shorter than 18 nt, or longer than 30 nt, then aligned with the *Glycine max* genome sequence downloaded from the Sequencing Resource website [62] using software named Soap [63]. The matched sequences were queried against non-coding RNAs from Rfam database [64] and NCBI Genbank database [65] by BLAST (*E*-value ≤ 0.01). Sequence matching exons and introns of mRNA, rRNAs, tRNAs, scRNA snRNA and snoRNA were excluded from further analysis. The remaining unannotated sRNA sequences were searched against miRBase 19.0 to identify known miRNAs. We predicted novel miRNAs as follow: small RNA sequences that can be aligned with known miRNAs, mRNAs, rRNAs, tRNAs, snRNAs, snoRNAs and repeat sequences were excluded; all the remaining unannotated small RNAs were subjected to online Mireap software to obtain the miRNA precursor sequences and analyze hairpin structures and Dicer cleavage sites [66]; small RNAs of very low abundance (with total reads fewer than 20) were eliminated; the structures of miRNA precursors were predicted by Mfold software [43] and checked manually: the pre-miRNAs must form stem–loop hairpin structures; the candidate miRNAs and miRNA* should come from opposite stem-arms and must be entirely located in the arms of the hairpin; miRNA::miRNA* duplex mismatches were restricted to four or fewer; and size of bulges between miRNA and miRNA* should be restricted to less than 2 bases [67,68].

Conservation of novel miRNAs was analyzed using sequence similarity to other known miRNA in the miRBase database. BLASTn search with an *E*-value cutoff of 10 was employed to find the miRNA sequences against the central miRNA Registry Database [42].

### 3.4. Quantitative Real-Time PCR

Small RNAs were isolated from soybean nodules treated with 125 mM NaCl at 0, 1, 3, 6, 12, 24 h using a mirVana-miRNA Isolation kit (Ambion, Austin, TX, USA) according to the manufacturer’s instructions. First-strand cDNA synthesis was performed by miRcute miRNA first-strand cDNA synthesis kit (Ambion, Austin, TX, USA) with an adapter primer with oligo(dT), 5′-GCGAGCACAGAATTAATACGACTCACTATAGGT12 (A, G, or C) (A, G, C, or T)-3′. Quantitative real-time PCR was performed on ABI PRISM 7500 Real-Time PCR System (ABI, Los Angeles, CA, USA) as follow: the total volume of the PCR reaction was 20 μL containing 10 μL SYBR Premix Ex Taq, 2 μL first stand cDNAs, 0.2 μM of each primer, 0.4 μL ROX Reference DyeII and 6.8 μL ddH_2_O. The PCR mixtures were preheated at 95 °C for 30 s, followed by 40 cycles of amplification (95 °C for 5 s, 60 °C for 34 s). Forward primers for the selected miRNAs and a commonly used reverse primer with the adapter sequence were included in Table S9. The results of qRT-PCR were analyzed using the 7500 system software. Each experiment contained three biological replicates and the statistical analysis of qPCR experiments performed by DPS (Data Processing System; International Business Machines Corporation: Armonk, NY, USA).

### 3.5. Target Prediction and Experimental Validation

We predicated the target genes of miRNAs using online miRU software [69]. The mismatches between the query miRNAs and the putative target must follow this criteria: one mismatch in the region complementary to nucleotides 2–12 from 5′ end of the miRNA allowed, but not at the 10th and 11th nucleotides, and three additional mismatches between 12- and 21-nucleotide positions allowed, but with no more than two continuous mismatches in this region [70].

For experimental validation, total RNA was extracted from mature nodules, and ployA mRNAs were isolated using a polyA Tract mRNA isolation system III (Promega, Madison, WI, USA). PolyA mRNAs were then subjected to a 5′-RACE reaction using a GeneRacer Kit (Invitrogen, Carlsbad, CA, USA) omitting calf intestinal phosphatase and decapping reaction treatments according to the instructions. For each candidate target, two reverse gene specific primers (GSP) were designed. Nested PCR were performed to amplify cleaved transcripts with reverse primers and GeneRacer adaptor-specific primers. The specific primers for the putative targets were shown in Table S9. The PCR products were subsequently cloned into pMD19-T Vector (Takara, Japan) for sequencing.

## 4. Conclusions

Using deep sequencing, we identified many previously unknown small RNAs that are expressed in mature nodules, and nodule miRNAs that are differentially regulated during salt stress. This approach also allowed us to estimate the expression abundance of each miRNA, revealing a dynamic regulation pattern of miRNAs during salt stress in functional nodules in soybean. We show that miRNAs target many functionally diverse genes involved in various biological processes. Further characterization of the highly expressed miRNAs in mature nodules and the miRNAs more responsive to salt stress will help to elucidate the molecular mechanisms of controlling nodule development, nitrogen fixation and salt tolerance of functional nodules in soybean.

## Figures and Tables

**Table 1:** Prediction of target genes for some nov-miRNAs and alignment to their target genes.

Name	Target gene	Function	Alignment between target and miRNA	Expectation (E)
gly_1	Glyma17g37430	B-Box Zinc Finger	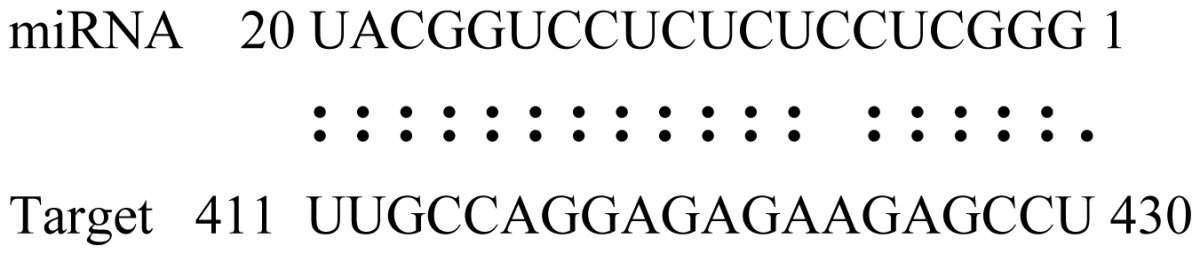	3.0
	Glyma04g03110	Thioredoxin-Related	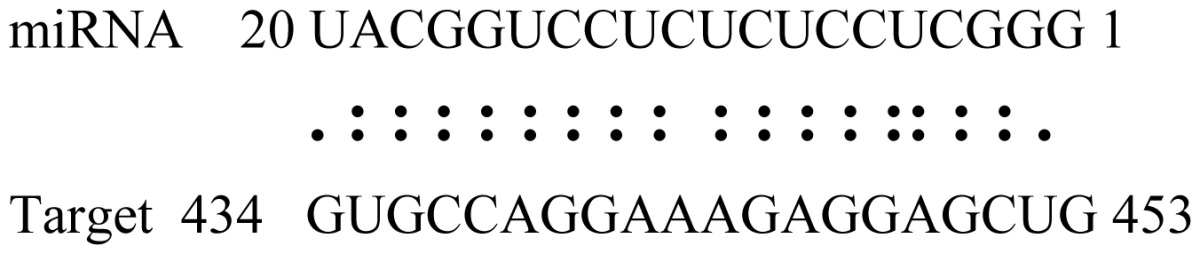	3.0
	Glyma15g06000	Glucosyl/Glucuronosyl Transferases	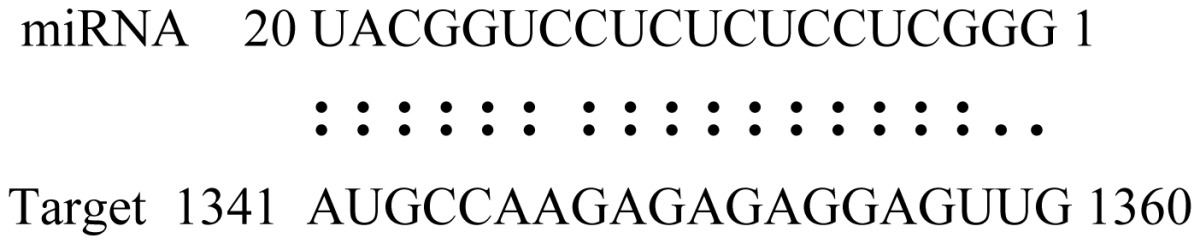	3.0
gly_3	Glyma09g37130	Lipoate-Protein Ligase	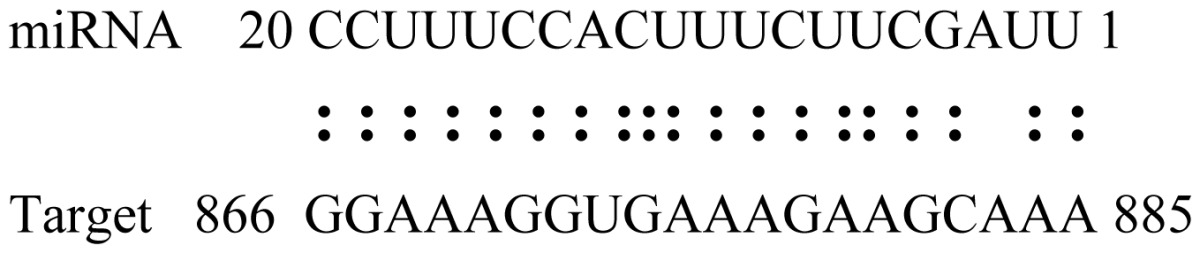	1.5
	Glyma16g27790	PPR Repeat Pentatricopetide Repeat-Containing Protein	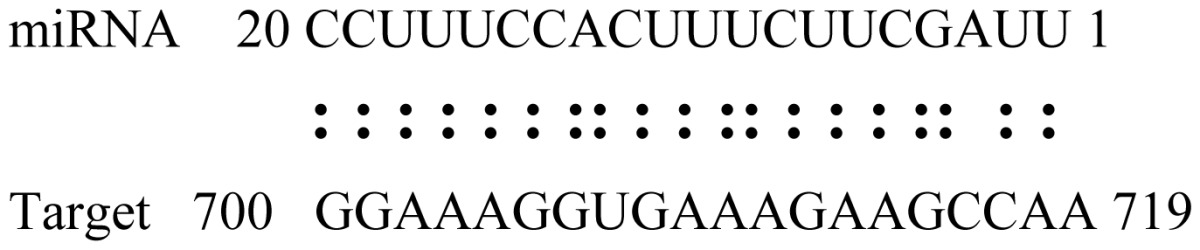	1.5
gly_5	Glyma10g34820	Drug Transporter-Related	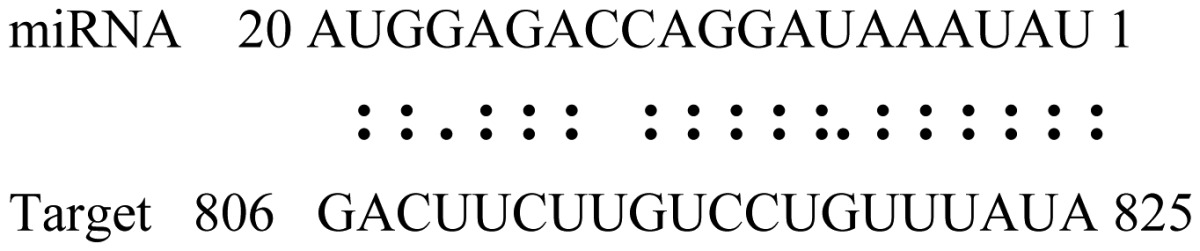	3.0
gly_6	Glyma02g05090	Lupus La Ribonucleoprotein	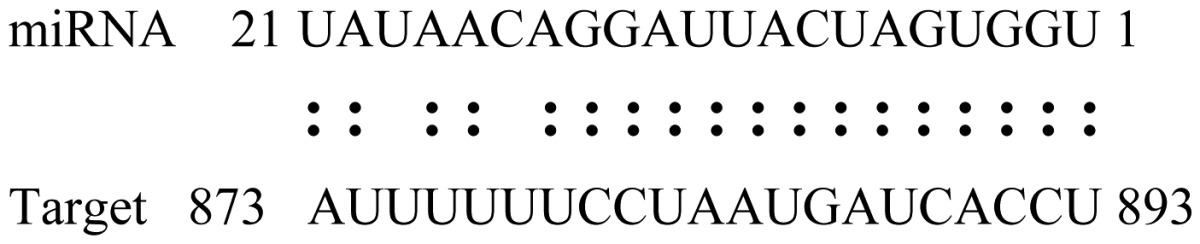	3.0
gly_7	Glyma19g45260	K+ Potassium Transporter	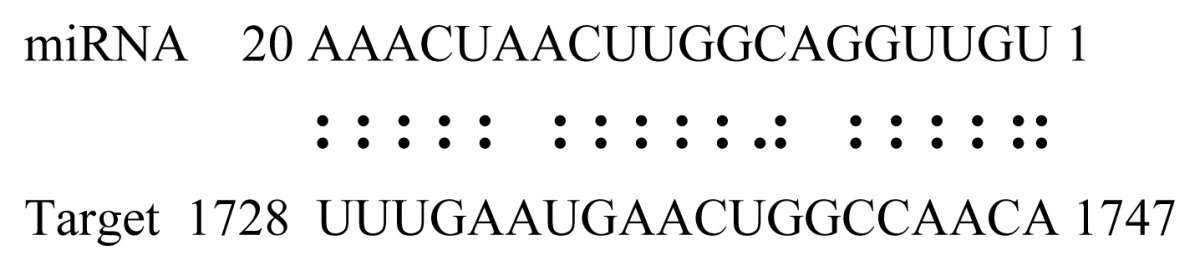	3.0
gly_10	Glyma04g43550	POT Family	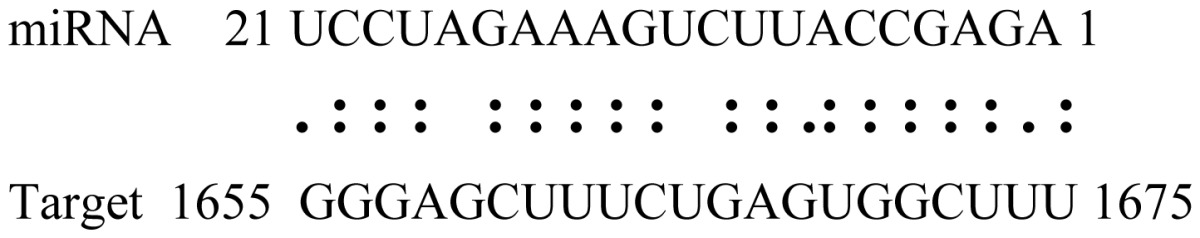	3.0
	Glyma07g40110	Protein Tyrosine Kinase	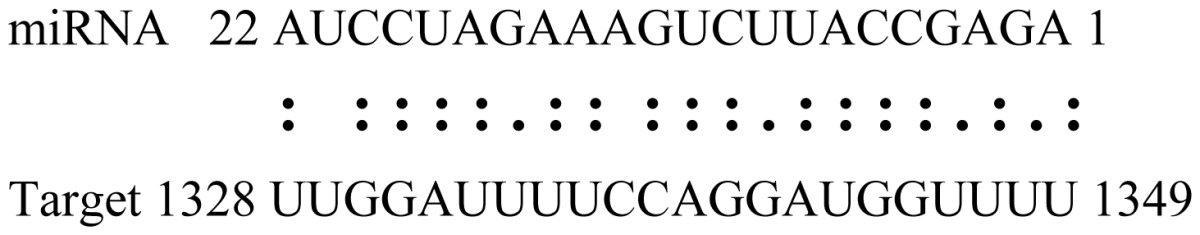	3.0
	Glyma09g37070	RNA-Binding Protein Related	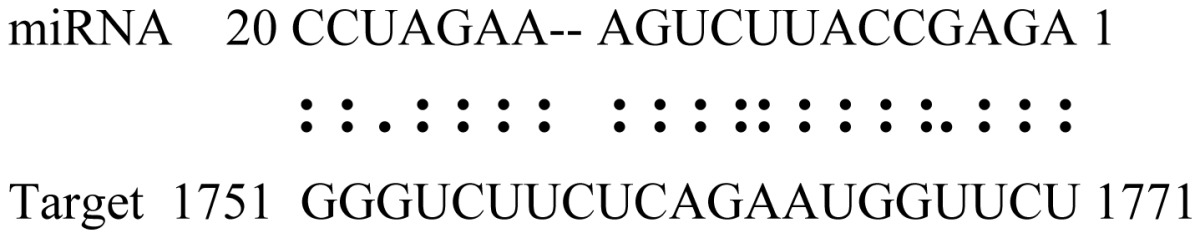	3.0
gly_11	Glyma05g02970	Ribosomal Protein S21	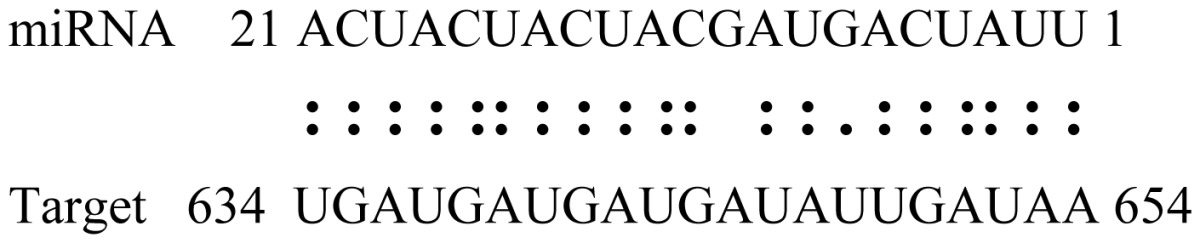	2.5
	Glyma08g11770	Ubiquitin-Like Protein Sumo/Smt3-Related	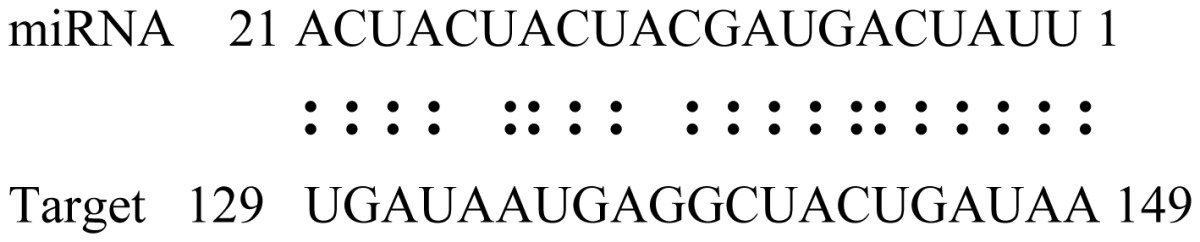	2.5
	Glyma13g17650	CREG1 Protein	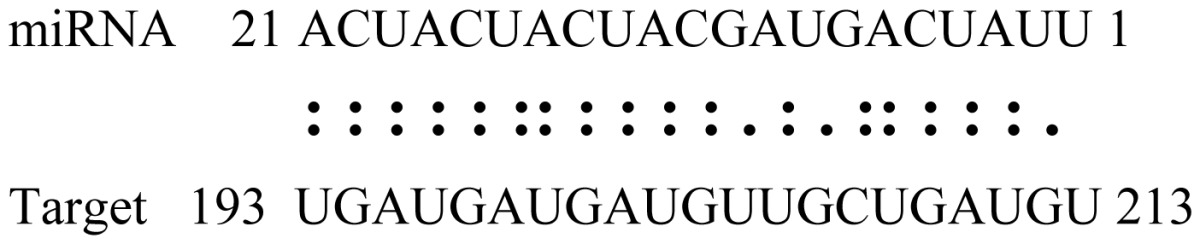	2.5
gly_12	Glyma09g30540	Aldose-1-Epimerase	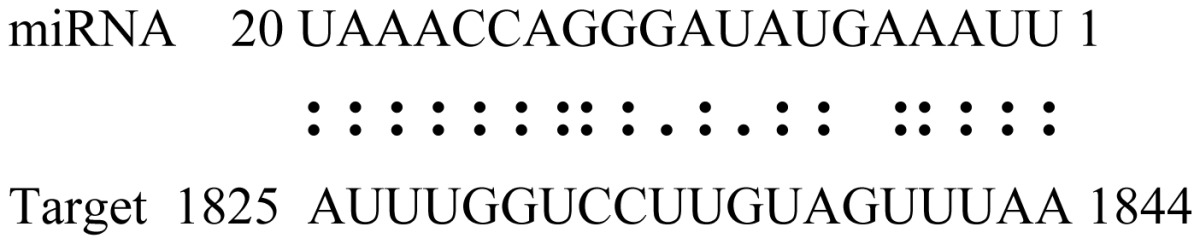	2.5
gly_32	Glyma13g24580	Mitochondrial Carrier Protein Related	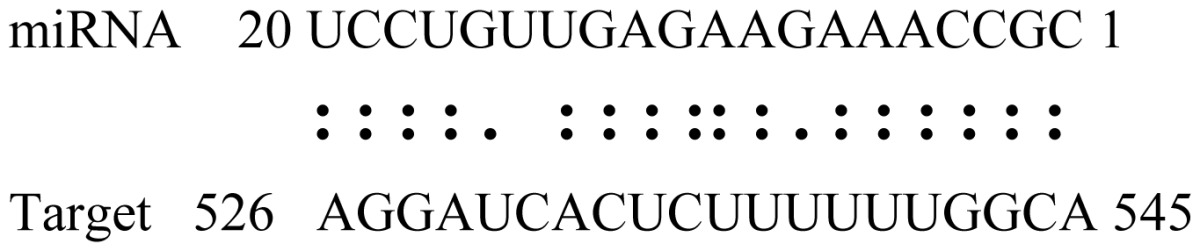	3.5
	Glyma04g05250	Hemoglobinase Family Member	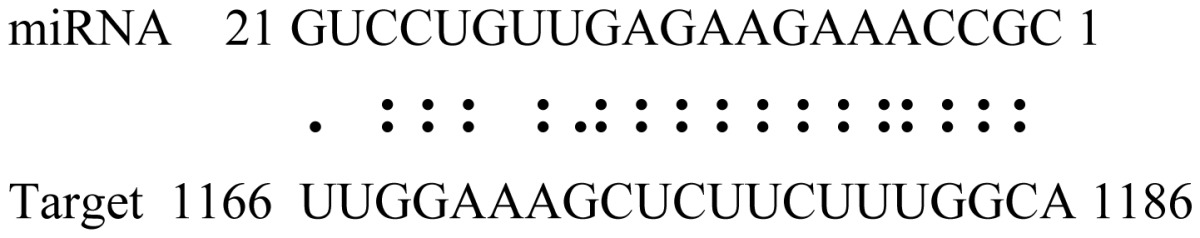	3.5
gly_37	Glyma08g05740	Eukaryotic Translation Initation Factor 3, Subunit 8 (Eif3s8)-Related	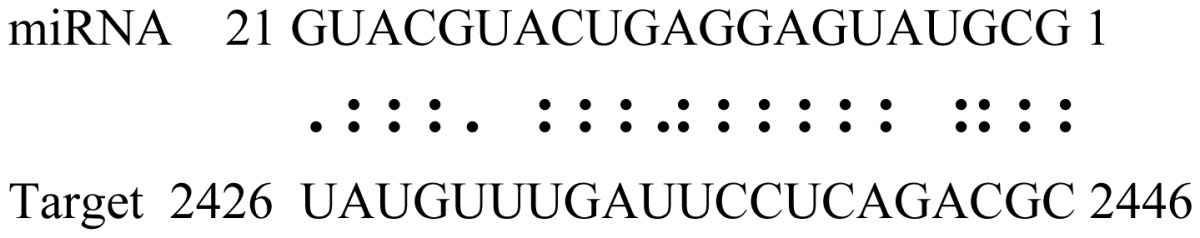	2.5
miR171p	Glyma11g17490	GRAS	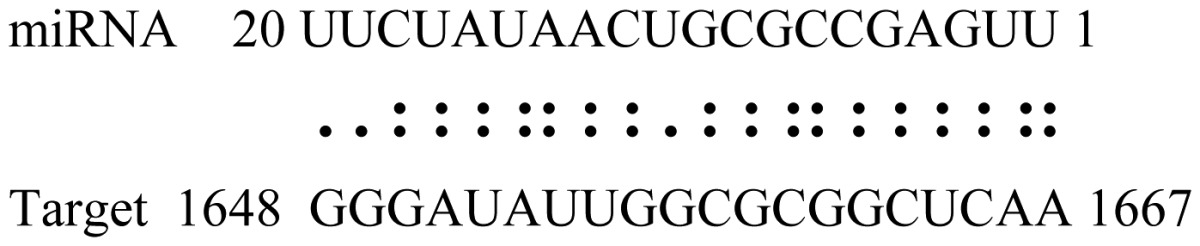	2.0
	Glyma20g03210	Glycoside Hydrolases	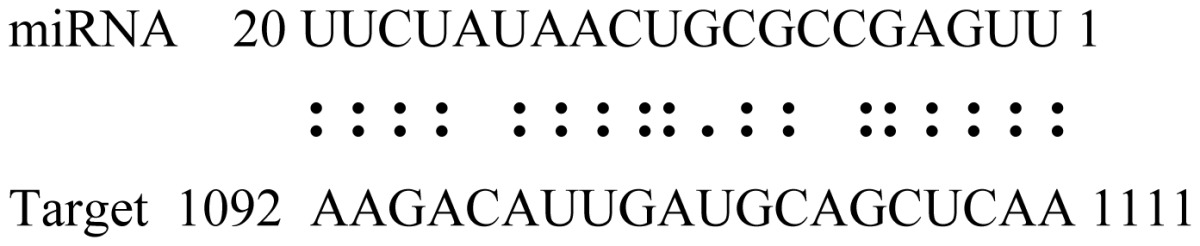	2.0
miR393i	Glyma19g27280	AFB2 Like Protein	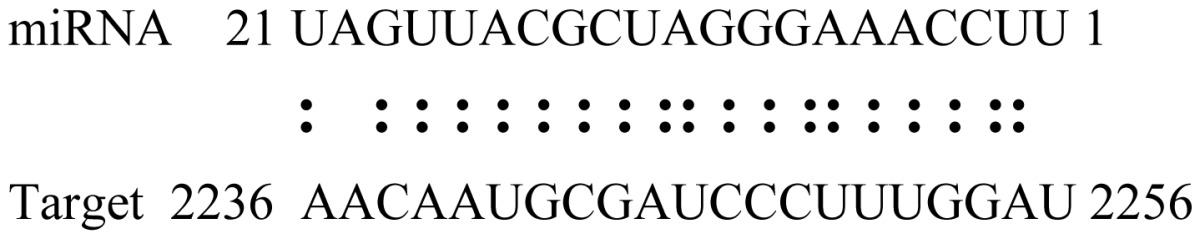	1.0
	Glyma19g39420	TIR1 Like Protein	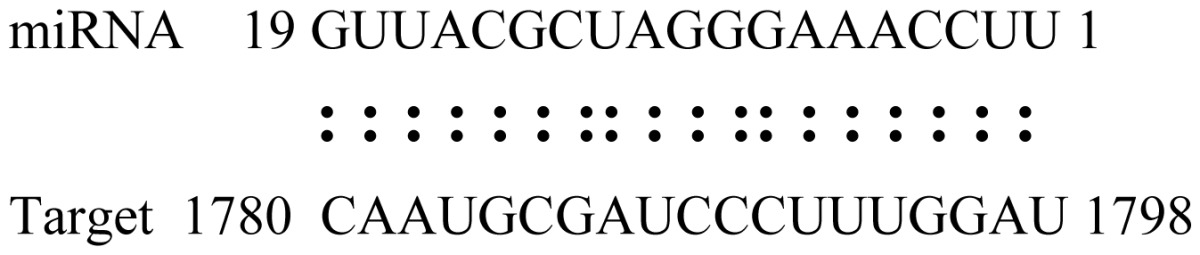	1.0

## Supplementary Files

Supplementary File 1 Supplementary Information (PDF, 169 KB)

Supplementary File 2 Supplementary Table 1 (XLS, 19 KB)

Supplementary File 3 Supplementary Table 2 (XLS, 28 KB)

Supplementary File 4 Supplementary Table 3 (XLS, 34 KB)

Supplementary File 5 Supplementary Table 4 (XLS, 27 KB)

Supplementary File 6 Supplementary Table 5 (XLS, 28 KB)

Supplementary File 7 Supplementary Table 6 (XLS, 23 KB)

Supplementary File 8 Supplementary Table 7 (XLS, 28 KB)

Supplementary File 9 Supplementary Table 8 (XLS, 151 KB)

Supplementary File 10 Supplementary Table 9 (XLS, 20 KB)
