# Supplementary material for: Identification and Dynamic Regulation of microRNAs Involved in Salt Stress Responses in Functional Soybean Nodules by High-Throughput Sequencing
Source: Int J Mol Sci. 2013 Jan 28;14(2):2717–38. doi: 10.3390/ijms14022717 (PMC3588011; doi:10.3390/ijms14022717)
Supplement: Supplementary File 1 — Supplementary Information (PDF, 169 KB) [file ijms-14-02717-s001.pdf]

Supplementary Information

Figure S1. RNA hairpin structure predictions for the novel miRNA precursors. Precursor structures were predicted by Mfold online.

> gly_1   Gm20:38248017:38248148:-

TAGTGAGCAAATCAGCAATAGGGCTCCTCTCTCCTGGCATGCAGACAGACATTATTTTGGTAGTTACCTACCAAAAGTTGGTATTAACAACATGCCAAGGGAGAGTTGCCCTGTGGCTGCTTTAGCTTAATC

```
Initial ΔG = -61.20
UAG     -   U    A       UC       C       CAGACAG-|  AUUA         U
   UGAGC AAA CAGC AUAGGGC  CUCUCUC UGGCAUG         AC    UUUUGGUAG U
   AUUCG UUU GUCG UGUCCCG  GAGAGGG ACCGUAC         UG    AAAACCAUC A
CUA     A   C    G       UU       A      AACAAUUA^  GUUG         C
```

> gly_2   Gm12:35489086:35489194:+

ACATGACAAATTAAGGTATTGGCGTGCCTCAATTTGAATACATGGCTATTATGACAAATCCAGCCTTGTAGTTTGATTGAGCCGCGTCAATATCTTATTTTGCTCTTCT

```
Initial ΔG = -41.90
ACAU  -|  U               C       UGAA    U   AUUAUG
    GA CAAA UAAGGUAUUGGCGUG CUCAAUU    UACA GGCU      A
    CU GUUU AUUCUAUAACUGCGC GAGUUAG    AUGU CCGA      C
UCUU  C^  U               C       UUUG    U   CCUAAA
```

> gly_3   Gm17:14170511:14170612:-

AACTGAGAGTGTGAGAGGTGAAGGAAGCTAATCTTGACCATTCAATCTTAATAGGGATTCGGTGTTCGTGATTAGCTTCTTTCACCTTTCCCACTTTCACCT

```
Initial ΔG = -55.00
AAC|     U                     U    C   UC      A
   UGAGAGUG GAGAGGUGAAGGAAGCUAAUC UGA CAU  AAUCUU A
   ACUUUCAC CUUUCCACUUUCUUCGAUUAG GCU GUG  UUAGGG U
UCC^     C                     U    U   GC      A
```

> gly_4   Gm07:5393976:5394157:-

ACCCAAGTTGGAGCTCTCAGCACTCCAGTGTTGAAGGAAGTGATGGTAAACCCTAGCTGCTAATTCATGGTTACCTTTGGTTTCACATAAGGAAGCATGTGAAGTCATGGGTCTGCATGAACTTGGAGATGGGGTTGCCTTGATCTTTTAGTATTGGAGTGAAGGGAGCTCCTAATTAGGCC

```
Initial ΔG = -82.50
AC  A    -|         AG                    AGUGAU     A     G   G    -       GUU     U  GU       AAG
  CC AGUU  GGAGCUCUC  CACUCCAGUGUUGAAGGA       GGUAA CCCUA CU CUAA UUCAUG   ACCU UG  UUCACAU    G
  GG UUAA  CCUCGAGGG  GUGAGGUUAUGAUUUUCU       CCGUU GGGGU GA GGUU AAGUAC   UGGG AC  AAGUGUA    A
CC  A    U^         AA                     AGUU--     -     A -    C       GUC     U  UG       CGA
```

> gly_5a   Gm10:893455:893562:+

AATAAATGGCACTACCTCTGCTCCTATTTTTATAAGAAACAAGTTATATAGTGTAAAATACACTACATCTTGTTTCTTATAAATAGGACCAGAGGTAGTATTAATTAT

```
Initial ΔG = -47.10
A|   A    C           C       UU              UUAUA       A
 AUAA UGG ACUACCUCUG UCCUAUU  UAUAAGAAACAAG      UAGUGUA  \
 UAUU AUU UGAUGGAGAC AGGAUAA  AUAUUCUUUGUUC      AUCACAU  A
-^   A    A           C       --              UAC--       A
```

>gly_5b   Gm13:37718475:37718554:+

TTACTATAATACTACCTATGGTCCTATTTATAAGAAACAAGTTGTAGTGTATAAATAGGACCAGAGGTAGTAATAAAGTA

```
Initial ΔG = -35.10
U|   A  A         A                 AGAA  AA
 UACU UA UACUACCU UGGUCCUAUUUAUA    AC    \
 AUGA AU AUGAUGGA ACCAGGAUAAAUAU    UG    G
-^   A  A         G                 GUGA  UU
```

> gly_6   Gm16:2804159:2804348:-

```
TACTCCTTCATGGTGATCATTAGGACAATATTTCATATATAGAAGCTTTCTTTCAATCATTATATATTTGTATAACAAAAGCCAAAAATGA
AGCAACACCTCCCACAATAAGCATGACACAAATTATAATTGGAAGAAAGCTTCTGTTATGTATTTGAAGTATTGTCCCAACGATCACCATG
AAAACAAT
Initial ΔG = -64.00
UACUCC            A  A             ----   -                 AUC      U      .-AUAACAAAAGC| A
      UUCAUGGUGAUC UU GGACAAUAUUUCA    UAU AUAGAAGCUUUCUUUCA   AUUAUA AUUUGU            CA A
      AAGUACCACUAG AA CCUGUUAUGAAGU    GUA UGUCUUCGAAAGAAGGU   UAAUAU UAAACA            GU A
UAACAA            C  C             UUAU   U                 ---      -      \ -----------^ A
                                                                         100~110
```

```
> gly_7    Gm05:41564645:41564756:+
GTTGGAGGAATACATGGGGATGTTGGACGGTTCAATCAAATCAAATCTCCTAATGGCTGGGTCCTTTGGTATGATTGAGCCGTGCCAATAT
CAAATCCTGCTTAACATGAAT
Initial ΔG = -42.40
------   -  -|   AUACAUGGG        -            A      UCU    AU
      GUU GG AGGA         GAUGUUGG ACGGUUCAAUCA AUCAAA   CCUA  \
      CAA UC UCCU         CUAUAACC UGCCGAGUUAGU UGGUUU   GGGU  G
UAAGUA   U  G^   AAA------        G            A      CCU    CG
```

```
> gly_8   Gm05:30104428:30104503:-
AGCAACCTATAGGCAATCTGTATGGACATATGGATCAGCAATCCATATGAACCATACGGATTGTCAATTTGTAAGT
Initial ΔG = -32.60
AGCAACCUAUA              A-|         CA
           GGCAAUCUGUAUGG   CAUAUGGAU   \
           CUGUUAGGCAUACC   GUAUACCUA   G
UGAAUGUUUAA              AA^         AC
```

```
> gly_9   Gm15:20061038:20061157:-
GTTTAGAGACCAACCCGTCTTAGAATGAGCACAATGTAGAATTGTGCTTATTCTACATCAGTTGTGGCGACGAACCAATGTAGAATTGTGC
TTATTCTAAAACGGGTTGGTCTCTAAACC
Initial ΔG = -65.10
-                  C              .-AU|  GA
 GUUUAGAGACCAACCCGU UUAGAAUGAGCACA    GUA  A
 CAAAUCUCUGGUUGGGCA AAUCUUAUUCGUGU    CGU  U
C                  A              \ --^  GU
```

```
> gly_10   Gm13:32275832:32275962:-
CAAAAGGATTTTAGGTCTTTCAACATGGCTCTTGCAATGGCTATTGGAGAAAGAGGAACACAATTGTCATGCGGACAAAAGCTGTGCATAA
CCATTGCAAGAGCCATTCTGAAAGATCCTAGAATCCTTTT
Initial ΔG = -60.20
C              -      AC                    | A  GG
 AAAAGGAUUUUAGG UCUUUCA  AUGGCUCUUGCAAUGG--CU UU  \
 UUUUCCUAAGAUCC AGAAAGU  UACCGAGAACGUUACC  GA AA  A
-             U       CU                  \^ G  AG
```

```
> gly_11a   Gm14:13819007:13819089:+
TCGTAGTTATTTATCAGTAGCATCATCATCAAAGTTCTTTACATGAAAGTTTGATGATGATGTTACCGATAAAGAGCTACGAG
Initial ΔG = -42.70
-        A      A                G-|   UUU
 UCGUAGUU UUUAUC GUAGCAUCAUCAUCAAA   UUC   \
 AGCAUCGA AAAUAG CAUUGUAGUAGUAGUUU   AAG   A
G        G      C                GA^   UAC
```

```
> gly_11b   Gm07:10004527:10004609:+
TCGTAGTTATTTATCAGTAGCATCATCATCAAAGTTCTTAACATAAAAGTTTGATGATGATGTTACCGATAAAGAGCTACGAG
Initial ΔG = -40.60
-|        A      A                  G CUUA
  UCGUAGUU UUUAUC GUAGCAUCAUCAUCAAA UU    A
  AGCAUCGA AAAUAG CAUUGUAGUAGUAGUUU AA    C
G^        G      C                  G AAUA
```

```
 > gly_11c   Gm07:10001915:10001997:+
TCGTAGTTATTTATCAGTAGCATCATCATCAAAGTTCTTTACATGAAAGTTTGATGATGATGTTACCGATAATGAGCTACGAG
Initial ΔG = -41.10
```

```
-       AU     A                 G-|  UUU
 UCGUAGUU UUAUC GUAGCAUCAUCAUCAAA   UUC   \
 AGCAUCGA AAUAG CAUUGUAGUAGUAGUUU   AAG   A
G       GU     C                 GA^  UAC

>gly_12   Gm19:37100978:37101075:+
TCATATTGATCAATTTGATTCCTTTACTTTTAAAAATAAGTTGATTTAGTCCTCGTTCCTATTTTTTTTAAAGTATAGGGACCAAATTAAT
CAATGTG
Initial ΔG = -26.40
U         C      A     U      U--       AGU-|   UUU
 CAUAUUGAU AAUUUG UUCCU UACUUU   AAAAAUA      UGA   A
 GUGUAACUA UUAAAC AGGGA AUGAAA   UUUUUAU      GCU   G
-         A      C     U      UUU       CCUU^   CCU

>gly_13a   Gm19:3610433:3610530:+
TCCATATCCACTTAAATGGATTGGATCTTGAATCCAAATTGTTTTTTAAACAAATGTGGATATAGATTTAAGATCCAATCCATTTAAGTAT
ATATGAA
Initial ΔG = -42.00
UC     CC                     ------     AA-|     U
  CAUAU  ACUUAAAUGGAUUGGAUCUUGA      AUCCA   UUGUUU \
  GUAUA  UGAAUUUACCUAACCUAGAAUU      UAGGU   AACAAA U
AA     UA                     UAGAUA     GUA^     U
>gly_13b   Gm17:32044297:32044393:-
TTTATATACACTTAAATGGATTGGATCTTGAATCCAAATTGTTTTAAAAAAATAGAGATATAGATTTAAGATCCAATCTATTTAAGTATAT
ATGGAT
Initial ΔG = -39.90
-|       C                         CAAAU      AAAA
 UUUAUAUA ACUUAAAUGGAUUGGAUCUUGAAUC      UGUUUU     \
 AGGUAUAU UGAAUUUAUCUAACCUAGAAUUUAG      AUAGAG     A
U^       A                         AU---      AUAA
>gly_13c   Gm15:43239794:43239891:+
TCCATATCCACTTAAATGGATTGGATCTTGAATCCAAATTGTTTTTTTTTTAATATGGATATAGATTTAAGATCCAATCCATTTAAGTATA
TATGGAT
Initial ΔG = -44.20
 -      CC                        ------|    A    UUUU
 UCCAUAU  ACUUAAAUGGAUUGGAUCUUGA          AUCCA AUUG    \
 AGGUAUA  UGAAUUUACCUAACCUAGAAUU          UAGGU UAAU    U
U       UA                        UAGAUA^    A    UUUU
>gly_13d   Gm14:27129224:27129321:+
TTCATATCCACTTAAATGGATTGGATCTTGAATCCAAATTGTTTTTTAAAAAATATGGATATAGATTTAAGATTCAATCCATTTAAGTAGA
TATGGAT
Initial ΔG = -42.60
-        C                         CAAAU---|      U
 UUCAUAUC ACUUAAAUGGAUUGGAUCUUGAAUC         UGUUUU U
 AGGUAUAG UGAAUUUACCUAACUUAGAAUUUAG         AUAAAA A
U        A                         AUAUAGGU^      A
>gly_13e   Gm12:37094765:37094863:-
TCTATATCCACTTAAATGGATTGGATCTTGAATCCAAATTGTTTTTAAAAAAAATATGGATATAGATTTAAGATCCAATCCATTTAAGTAG
ATATGGAT
Initial ΔG = -46.10
-        C                         CAAAU---|       A
 UCUAUAUC ACUUAAAUGGAUUGGAUCUUGAAUC         UGUUUUU \
 AGGUAUAG UGAAUUUACCUAACCUAGAAUUUAG         AUAAAAA A
U        A                         AUAUAGGU^       A
      90        80        70        60        50
>gly_13f   Gm11:35031000:35031097:-
TACATATCCACTTAAATGGATTGGATCTTGAATCCAAATTATTTTTGAAAAAAATATGGATATAGATTTCAGATCCAATCCATTTAAGTTG
ATATGGA
Initial ΔG = -42.40
```

```
UA      C                   U    CAA    AU--|   G
  CAUAUC ACUUAAAUGGAUUGGAUCU GAAUC  AUU     UUUU A
  GUAUAG UGAAUUUACCUAACCUAGA UUUAG  UAG     AAAA A
AG      U                  C     AUA    GUAU^   A
```

>gly_13g Gm10:4635902:4636116:+

ATATGCCAAATTCATATCCACTTAAATGGATTGGATCTTGAATCCAAATTATTTTTAAAAAAATGTGAATATAAATATAAGATTCAATTTA
TTTAAGTAGATATGGATTGAATTCTTAAAAATCCAATCCATGTCCACCCCTGGTGACGAGATAATGTCCCTTAAGAAGGCTTTTCAAGATC
CAATCCATTTAAGTAGATATGAATTTGGTTAGA

Initial ΔG = -79.10

```
AUAU             C                       U--  .-AAAUUAUU|     A    G     AUAAA
   GCCAAAUUCAUAUC ACUUAAAUGGAUUGGAUCUUGAA   CC           UUUAAA AAAU UGAAU     \
   UGGUUUAAGUAUAG UGAAUUUACCUAACCUAGAACUU   GG           GAAUUU UUUA ACUUA     U
AGAU             A                       UUC  \ --------^     A    -     GAAUA
```

>gly_13h Gm07:13683421:13683518:-

TCCATATCCACTTAAATGGATTGGATCTTGAATCCAAATTGCTTTTTAAAAAAATATGGATATAGATTTAAGATCCAATTCATTTAAGTGG
ATATGAA

Initial ΔG = -46.20

```
UC                            ------|    A   GCUUU
  CAUAUCCACUUAAAUGGAUUGGAUCUUGA      AUCCA AUU    U
  GUAUAGGUGAAUUUACUUAACCUAGAAUU      UAGGU UAA    U
AA                            UAGAUA^    A   AAAAA
```

>gly_13i Gm07:42931413:42931510:+

TCCATATCCACTTAAATGGATTGGATCTTGAATCTAAATTGTTTTTAAAAAAAATTGAATATAGATTCAAGATCCAATCTATTTAAGTAGA
TATGGAT

Initial ΔG = -51.50

```
-        C                           A   --|     A
 UCCAUAUC ACUUAAAUGGAUUGGAUCUUGAAUCUA AUU  GUUUUU  \
 AGGUAUAG UGAAUUUAUCUAACCUAGAACUUAGAU UAA  UAAAAA  A
U        A                           A  GU^      A
```

>gly_13j Gm06:39048243:39048358:-

TATGACCAAATCCATATCCACTTAAATGGATTGGATCTTGAATTCAAATTATTTTAAAAAAATATAGATATAGATTCAAGATCCAATCCAT
TTAAGTAGATATGGATTGGATGTGG

Initial ΔG = -53.20

```
UAUGA-|   A        C                        CAAAU    UAAAA
      CCAA UCCAUAUC ACUUAAAUGGAUUGGAUCUUGAAUU    UAUUU     \
      GGUU AGGUAUAG UGAAUUUACCUAACCUAGAACUUAG    AUAGA     A
GGUGUA^   -        A                         AU---    UAUAA
```

>gly_13k Gm06:22728700:22728798:-

TCTATATCCACTTAAATGGATTGGATCTTGAATCCAAAGTGTTTAAAAAAAAAATATGGATATAGATTTAAGATCCAATCCATTTAAGTAG
ATGCGGAT

Initial ΔG = -43.50

```
-  UA    C                        CAAA---|     AAA
 UC  UAUC ACUUAAAUGGAUUGGAUCUUGAAUC      GUGUUU   \
 AG  GUAG UGAAUUUACCUAACCUAGAAUUUAG      UAUAAA   A
U  GC    A                        AUAUAGG^     AAA
```

>gly_13l Gm05:14960200:14960296:+

TTCATATCCACTTAAATGGATTGGATCTTGAATCCAAATTGATTTTAAAATATATGGATATAGATTTAAGATCCAATCCATTTAAGTAGAT
ATGGAT

Initial ΔG = -45.50

```
-|       C                         CAA  G  UUUA
 UUCAUAUC ACUUAAAUGGAUUGGAUCUUGAAUC  AUU AU   A
 AGGUAUAG UGAAUUUACCUAACCUAGAAUUUAG  UAG UA   A
U^       A                         AUA  G  UAUA
```

>gly_13m Gm05:4272932:4273029:+

TCCATATCCACTTAAATGGATTGGATCTTGAATCCAAATTGTTTTAAAAAAAATATGAATATAGATTTAAGATCCAATCCATTTAAGTAGA
TATGGAT

Initial ΔG = -48.60

```
 -       C                         CAAAU---|     A
 UCCAUAUC ACUUAAAUGGAUUGGAUCUUGAAUC        UGUUUU A
 AGGUAUAG UGAAUUUACCUAACCUAGAAUUUAG        AUAAAA A
U        A                         AUAUAAGU^     A
```

>gly_13n   Gm01:28289348:28289444:-

TCCATATCCACTTAAATGGATTGGATCTTGAATCCAAATTTTTTTAAAAAATATATGGATATAGATTTAAGATCCAATCCATTTAAGTAGA
TATGAA

Initial ΔG = -42.70

```
UC|      C                        CAA    UUU   AA
  CAUAUC ACUUAAAUGGAUUGGAUCUUGAAUC   AUUU   UA   \
  GUAUAG UGAAUUUACCUAACCUAGAAUUUAG   UAGG   AU   A
AA^      A                        AUA    UAU   AA
```

>gly_13o   Gm01:5733903:5733997:+

TCCATATCAACTTAAATGGATTGGATCTTGAATCCAATTTTTTTTTTAAATATGGATATAGATTTAAGATTCAAACCATTTAAGTAGATAT
GGAT

Initial ΔG = -39.40

```
-        A          A            ------     -|    UUU
 UCCAUAUC ACUUAAAUGG UUGGAUCUUGA       AUCCA AUUU    \
 AGGUAUAG UGAAUUUACC AACUUAGAAUU       UAGGU UAAA    U
U        A          A            UAGAUA     A^    UUU
```

>gly_14   Gm14:22838069:22838233:-

TAGAAGACTCCAAGTAGATTGAGCCAGAGATGCAAGAGAAGACCCTAGGGTTCTCATGAGCCTTAGGGCAGATTTTATGGCCATGGGCTAA
GTATGAGCCCACTTATCATTGTACATATTAGATTAAGGTTTCATTATTTTTGGGTCGTGTATTTAGGGCTCCAT

Initial ΔG = -50.80

```
UAGAA   CU   -     G U   G         CAA     .-AGA|            U
     GA   CC AAGUA A UGA CCAGAGAUG    GAGA      CCCUAGGGUUC  C
     CU   GG UUUAU U GCU GGUUUUUAU    CUUU      GGGAUUCCGAG  A
UAC--   CG   A     G -   G         UA-     \ ---^            U
```

70-127

>gly_15   Gm04:44568204:44568328:-

CTTTTTTAAATTTAAACAATATCCTGGAGATTTGAAATTAAATTTTAATTGAGGGTAGAATTATTGGGAAACAATTCAAATTTGTAATTTC
AAGTCTCTAGGATATTGTTTAGATTTGAAAAAGA

Initial ΔG = -54.00

```
-                                           |      AUU
 CUUUUUUAAAUUUAAACAAUAUCCUGGAGAUUUGAAAUUA--AAUUUUA   G
 GAAAAAGUUUAGAUUUGUUAUAGGAUCUCUGAACUUUAAU  UUAAGAU    A
A                                        \  ^       GGG
```

69-83

>gly_16   Gm03:45559347:45559504:-

GGTTCATACCAGAGAACATAAACCCTAACCCGGAAAGGATAGAATTCTTAGGGATAACGGAACAATAGGGGGAATCCCAAAAGCAGGACGC
AAATTAAAATCTTGAGCATAGAATTCTATTTCATTCCGGATTATGGTATATGTTCTTGAAAGTGAAC

Initial ΔG = -53.70

```
G     ACCA        A   C  C     A-           UA----     .-AACGGAACAAUA|    G
 GUUCAU   GAGAACAUA ACC UAA CCGGAA  GGAUAGAAUUCU      GGGAU              GGGG \
 CAAGUG   UUCUUGUAU UGG AUU GGCCUU  UUUAUCUUAAGA      UUCUA              CCCU  A
-     AAAG        A   U  A     AC           UACGAG     \ ------------^    A
```

80-98

>gly_17   Gm04:28532443:28532536:-

GCCAGTGAAATCGGTGCGAGTGTCTTCGCCTCTGAGAGAGATACTATGAGATCTCAAGCCTCGGAGGCGTAGATACTCACACCTCTTTTTC
TGG

Initial ΔG = -49.70

```
G   U    UC    C         U            A---|     ACU
 CCAG GAAA  GGUG GAGUGUCU CGCCUCUGAG      GAGAU    A
 GGUC UUUU  CCAC CUCAUAGA GCGGAGGCUC      CUCUA    U
-   U    CU    A         U            CGAA^     GAG
```

>gly_18   Gm04:33363384:33363625:+

TTTAACCACTCTCACTTCATAGTTTTTTCTGTATGATAACCAATTCTTGAGTATTTGTATCTATATGCATGACAACCTCTTCATAATATTC
TTGAGTATATTCATATCTATATATAAATGAGTCAAGATAATCAAATTAAATTAATAATAATAATAATAGTAATAATAATAAAAGCTTATGC
TGAATTCATCTTAATAATTGATCAGAAAACAGATAAAGCTATGAAGTGAGAATGGCTAAA

Initial ΔG = -54.80

```
U   A   C                   U      .-AUGAUAACCAAU      G-         CU--      .-C|    CAA
 UUA  CCA  UCUCACUUCAUAGUUUU  UCUGU                UCUUGA  UAUUUGUAU     AUAUG    AUGA    C
 AAU  GGU  AGAGUGAAGUAUCGAAA  AGACA                AGAACU  GUAAAUAUA     UAUAC    UACU    C
A   C   A                   U      \ ------------      GA         UAUC      \ -^    UCU
```

80-98

>gly_19   Gm04:46348842:46348957:+
GATTGAAGGGTTGCTGAAGCATCTGCTGACTCATTCATACACACAGAATAGTGATTCAAATTGCTAAGGTAATTGTGTGAATGAAGCAGGA
GATATTTTGCATCCCTCTTTCTTTG

Initial ΔG = -41.10

```
----   UU A    U    U   GC    G   AC            .-A|   AG
    GA   G AGGG UGC GAA  AUCU CUG  UCAUUCAUAC     CAC   A
    CU   C UCCC ACG UUU  UAGA GAC  AGUAAGUGUG     GUG   A
GUUU   UU -    U    U   A-    G   GA            \ -^   AU
```

55-73

>miR5037e   Gm18:61631517:61631620:-
AGTTTTTAACAACCCTCAAAGGCTTCCACTACTTCATATTTCATTCCAGTGAGAATATCACAAACATGAAAAAGTGGTGGAACTTTGAGGC
TTGTTGAAGCCTT

Initial ΔG = -41.10

```
-|  U           C        GC             CAUA      UCCA    GA
  AG UUUUUAACAA CCUCAAAG  UUCCACUACUU     UUUCAU    GUGA   \
  UC GAAGUUGUU  GGAGUUUC  AAGGUGGUGAA     AAAGUA    CACU   A
U^  C           C        --             ----      CAAA    AU
```

>gly_20   Gm18:54327830:54328015:+
GTTGCTTCCTGCACTTCCCAAGTTGCTTCCTGTACTCCTTCAGGAATATTTTTTTGACCTTCTGGGTTGGTCATTCCATAAGACAAAAAGA
GAATGCATTCAAGAAGGTAATTTTACATTTCGGATTGATCGTTCTGAAAAGTGAAAAGGGAGTGCAGGAAACAACTAGGAAATGCAGGAAG
CAAA

Initial ΔG = -79.40

```
G            C    CA    C                |       U
 UUGCUUCCUGCA UUCC  AGUUG UUCCUGUACUCCU--UCAGGAA  A
 AACGAAGGACGU AAGG  UCAAC AAGGACGUGAGGG  AGUUUUU  U
A            A    A-    A               \ ^       U
```

60-140

>gly_21a   Gm17:19846241:19846404:+
TTGTTCTTGACACTTATTTTTAGTCCCTAATAAATTATCAAATTTTGTGTTTGCTACATGATAAATATTTCTCATTTATTATTAATACAGA
CTAAAAAAATTTTCTAATTTGTTAGGAAGCAAACACAAAATTTTCTAATTTATCAGGGACTAAAACAAAACAT

Initial ΔG = -47.50

```
U    C   ACACUUAU           A         UC                 .-AC|          AUU
 UGUU UUG        UUUUAGUCCCU AUAAAUUA  AAAUUUUGUGUUUGCU      AUGAUAAAU    \
 ACAA AAC        AAAAAUCAGGGA UAUUUAAU  UUUAAAACACAAACGA      UAUUAUUUA    U
U    -   --------           C         CU                 \ --^          CUC
```

84-117

>gly_21b   Gm16:8096019:8096185:+
TGACACATTTTTTAGTTCTTGATAAATTAATGAATTTTGTTTTTGCTCCTTGATAATTTTTTTTCATTTGTGAGTGGTCCTTTTCAAAGG
GACAAATAACAAATGAAATTTTTATTAGGGAGCAAATACAAAATTTTCTAATTTATCAGGGACTAAAAGAAGTGTC

Initial ΔG = -79.80

```
U|     A                      AU         U                UUUUUU          GAGUG        U
 GACAC  UUUUUUAGUUCUUGAUAAAUUA  GAAUUUUGU UUUGCUCCUUGAUAA       UUUCAUUUGU      GUCCUUU  \
 CUGUG  AGAAAAUCAGGGACUAUUUAAU  UUUAAAACA AAACGAGGGAUUAUU       AAAGUAAACA      CAGGGAA  C
-^     A                      CU         U                UUU---          AUAAA        A
```

>gly_21c   Gm07:17786347:17786503:+
CCACTTATTTTTAGTTCTTGGTAAATTAGCAAATTTTATGTTTATTCTATAATAAATATTTCTCATTTATTATTAATACAGACAAAAATGA
AATTTGTTAATTTATTAAGAAGCAAATACAAAATTTTCTAATTTATCAGGGACTAAAATAAAATAT

Initial ΔG = -38.40

```
CCACUUAU                         C        A      A-     A        .-AUUUCUCAU|   U
```

```
         UUUUAGUUCUUGGUAAAUUAG AAAUUUU  UGUUU  UUCU  UAAUAAAU            UUA U
         AAAAUCAGGGACUAUUUAAUC UUUAAAA  AUAAA  AAGA  AUUAUUUA            AAU A
UAUAAAAU                     U       C      CG     -          \ ---------^   U
```

78-100

>gly_21d Gm05:9977544:9977700:-
ACACTTATTTTTAGCCTTTGATAAATTAGAAAATTTTGTGTTTGCTTCATGATAAATATTTCGCATATGTTATTAATCCAGACTAAAACAA
AATTTGCTAATTTATTAGGAAACAAACACAAAATATTCTAATTTATCAGGGACTAAAATAAAATAT
Initial ΔG = -46.00

```
ACACUUAU|     C                A            C    A        AUUUC    UA---     A    AUC
        UUUUAG CUUUGAUAAAUUAGAA AUUUUGUGUUUG UUC UGAUAAAU      GCA       UGUU UUA    C
        AAAAUC GGGACUAUUUAAUCUU UAAAACACAAAC AAG AUUAUUUA      CGU       ACAA AAU    A
UAUAAAAU^     A                A            A    G        AU---    UUAAA     -    CAG
```

>gly_22 Gm09:109565:109722:-
CAGGAAAATAAAATGAATTAAGCTTCTCCATAAGTTAAAATTAGCTTAAGCATAAGCTAATTCATAGAATTTCTCTCATTTAGCTTCTCCA
AAATCTAATTTTAACTTGGACATAAGCTAATTTAACTTATGGAAGAAGCTCAATTCATTTTTTCTTC
Initial ΔG = -53.30

```
C      AAU          A      -           A            .-AGCAU|       UUCAU    AU
 AGGAA   AAAAUGAAUU AGCUUC UCCAUAAGUU  AAAUUAGCUUA          AAGCUAA      AGA    \
 UUCUU   UUUUACUUAA UCGAAG AGGUAUUCAA  UUUUAAUCGAAU         UUCGAUU      UCU    U
C      ---          C      A           -            \ -----^       UAC--    CU
```

88-111

>miR1507d Gm09:16565920:16566038:-
CTTTTAAGATGATGTTTGCTAGAGGTGTTTGGGATGAGAGAATAGAATTTTTCAAATGCTTGAAAGTGATCTCTTCCCTCATTCCAAACA
TCATCTAACACACATGATGTTAACCAAC
Initial ΔG = -44.70

```
CUU--     G  G    U  C    -|                A   U    AUU        A
      UUAA AU AUGU UG UAGA  GGUGUUUGGGAUGAG GAA AGA    UUUUCAA  U
      AAUU UA UACA AC AUCU  CUACAAACCUUACUC CUU UCU    GAAGUU   G
CAACC     G  G    C  A    A^                C   C    AGU        C
```

>miR405b Gm04:34315950:34316105:-
CGGTTATTAGTAATAATCGTCTTAGAAAGTCAATGTTTTAAGACGGTTATTAAAAAACCGTTTTAAAAAGTTTGTAATGAAACACTTTTTA
AGATGGTTATTTAGTGATAATCGTCTTAAAAAATATATTTTCTAAGACGGTTATTACTGATAACC
Initial ΔG = -74.40

```
C                             CAAUG                AAA---|             UU  AA
 GGUUAUUAGUAAUAAUCGUCUUAGAAAGU     UUUUAAGACGGUUAUUA      AACCGUUUUAAAAAGU  GU   U
 CCAAUAGUCAUUAUUGGCAGAAUCUUUUA     AAAAUUCUGCUAAUAGU      UUGGUAGAAUUUUUCA  CA   G
-                             UAUAA                GAUUUA^             --  AA
```

>gly_23 Gm02:42141385:42141491:+
TTTCAATTTGTTTTCTGTTTCAAAGATTTGTACAGGAAACATTGAAAACAACTTTTTTTGTTCTCTGTTTCCTTTACAGATCTTTGGAAA
CAGGCAACAGATTGAA
Initial ΔG = -47.80

```
U           U       -|           C         UU  A      CUU
 UUCAAUUUGUU UCUGUUU  CAAAGAUUUGUA AGGAAACA  GA AACAA    \
 AAGUUAGACAA GGACAAA  GUUUCUAGACAU UCCUUUGU  CU UUGUU    U
-           C       G^           U         --  C      UUU
```

>gly_24 Gm10:2905320:2905422:-
AAAGTTGAGGGGAATGTCGTTTGGTTCGAGATCATTCATGCAAGTAGTCTCAGACATAACTCTTCTGAGTGATTTCGGACCAGGCTTCATT
CCCCTCAGCTAC
Initial ΔG = -57.20

```
AA               UC                      UGCA    A--|    U
  AGUUGAGGGGAAUG  GUUUGGUUCGAGAUCAUUCA     AGU      GUC  \
  UCGACUCCCCUUAC  CGGACCAGGCUUUAGUGAGU     UCA      CAG  C
CA               UU                      CUUC    AUA^    A
```

>gly_25a Gm18:29620793:29620900:-
AATATTTATAATTAGAAAACTATTTTATATGTTACTAATAATCAATAATAAATATATATATTTATTGATTATTAGTAACATATAAAATAGT
TTTCTAATTATAAATAT

```
Initial ΔG = -54.80
A|                                               UAAAUA
  AUAUUUAUAAUUAGAAAACUAUUUUAUAUGUUACUAAUAAUCAAUAA       \
  UAUAAAUAUUAAUCUUUUGAUAAAAAUAUACAAUGAUUAUUAGUUAUU       U
-^                                               UAUAUA

>gly_25b   Gm18:3402620:3402707:-
AATATTTATAATTAAAAAACTATTTTATATCTTAATAATAATCAATAAATATTAGTAACATATAAAATAGTTTTCTAATTATAAATAT
Initial ΔG = -25.50
A|              A               C    A      AUCA
  AUAUUUAUAAUUA AAAACUAUUUUAUAU UUA UAAUA     \
  UAUAAAUAUUAAU UUUUGAUAAAAAUAUA AAU AUUAU     A
-^             C                C    G      AAAU
>gly_25c   Gm18:29620792:29620899:+
AATATTTATAATTAGAAAACTATTTTATATGTTACTAATAATCAATAAATATATATATTTATTATTGATTATTAGTAACATATAAAATAGT
TTTCTAATTATAAATAT
Initial ΔG = -54.60
A|                                               A  UAU
  AUAUUUAUAAUUAGAAAACUAUUUUAUAUGUUACUAAUAAUCAAUAA UA    A
  UAUAAAUAUUAAUCUUUUGAUAAAAAUAUACAAUGAUUAUUAGUUAUU AU    U
-^                                               -  UUA
>gly_25d   Gm06:45100817:45100917:-
AATATTTATAATTAGAAAACTATTTTATTTGTTACTAATAATCAATAAATATATTTATTGATTATTAGTAACATATAAAATAGTTTTCTAA
TTATAAATAT
Initial ΔG = -52.20
A|                           U                     U
  AUAUUUAUAAUUAGAAAACUAUUUUAU UGUUACUAAUAAUCAAUAAA A
  UAUAAAUAUUAAUCUUUUGAUAAAAAUA ACAAUGAUUAUUAGUUAUUU U
-^                           U                     A
>gly_25e   Gm10:6636180:6636267:-
AATATTTATAATTAGAAAACTATTTTATATCTTACTAATAATCAATAAATATTAATAACATATAAAATAGTTTTCTAATTATAAATAT
Initial ΔG = -31.70
A|                              C    C      AUCA
  AUAUUUAUAAUUAGAAAACUAUUUUAUAU UUA UAAUA     \
  UAUAAAUAUUAAUCUUUUGAUAAAAAUAUA AAU AUUAU     A
-^                              C    A      AAAU
>gly_25f   Gm15:37277120:37277207:-
AATATTTATAATTAGAAAACTATTTTACATCTTACTAATAATCAATAAATATTAGTAACATATAAAATAGTTTTCTAATTATAAATAT
Initial ΔG = -33.60
A|                          C  C          AUCA
  AUAUUUAUAAUUAGAAAACUAUUUUA AU UUACUAAUA     \
  UAUAAAUAUUAAUCUUUUGAUAAAAU UA AAUGAUUAU     A
-^                          A  C          AAAU
>gly_25g   Gm15:37421666:37421753:-
AATATTTATAATTAGAAAACTATTTTATATCTTACTAATAATCAATAAATATTAGTAACATATAAAATAGTTTTCTAATTATAAATAT
Initial ΔG = -37.70
A|                              C         AUCA
  AUAUUUAUAAUUAGAAAACUAUUUUAUAU UUACUAAUA      \
  UAUAAAUAUUAAUCUUUUGAUAAAAAUAUA AAUGAUUAU      A
-^                              C         AAAU
>gly_25h   Gm15:37480614:37480701:-
AATATTTATAATTAGAAAACTATTTTATATCTTACTAATAATCAATAAATATTAGTAACATATAAAATAGTTTTCTAATTATAAATAT
Initial ΔG = -37.70
A|                              C         AUCA
  AUAUUUAUAAUUAGAAAACUAUUUUAUAU UUACUAAUA     \
  UAUAAAUAUUAAUCUUUUGAUAAAAAUAUA AAUGAUUAU     A
-^                              C         AAAU

>gly_25i   Gm15:37461824:37461911:-
AATATTTATAATTAGAAAACTATTTTATATCTTACTAATAATCAATAAATATTAGTAACATATAAAATAGTTTTCTAATTATAAATAT
```

Initial ΔG = -37.70
```
A|                             C         AUCA
  AUAUUUAUAAUUAGAAAACUAUUUUAUAU UUACUAAUA    \
  UAUAAAUAUUAAUCUUUUGAUAAAAAUAUA AAUGAUUAU    A
-^                             C         AAAU
```
>gly25j   Gm17:12625806:12625906:+

AATATTTATAATTAGAAAACTATTTTATATGTTACTAATAATCAATAAATATATTTATTGATTATTAGTAACATATAAAATAGTTTTCTAA
TTATAAATAT

Initial ΔG = -56.10
```
A|                                              U
  AUAUUUAUAAUUAGAAAACUAUUUUAUAUGUUACUAAUAAUCAAUAAA A
  UAUAAAUAUUAAUCUUUUGAUAAAAAUAUACAAUGAUUAUUAGUUAUUU U
-^                                              A
```
>gly_25k   Gm17:12625807:12625907:-

AATATTTATAATTAGAAAACTATTTTATATGTTACTAATAATCAATAAATATATTTATTGATTATTAGTAACATATAAAATAGTTTTCTAA
TTATAAATAT

Initial ΔG = -56.10
```
A|                                              U
  AUAUUUAUAAUUAGAAAACUAUUUUAUAUGUUACUAAUAAUCAAUAAA A
  UAUAAAUAUUAAUCUUUUGAUAAAAAUAUACAAUGAUUAUUAGUUAUUU U
-^                                              A
```

>gly_26   Gm20:26233269:26233370:-

CATATTCATCACCAGATGAATTCACACTAACATGGGTCAGCAATCTCCTTTCTTATGGTTGTTACTCTCATGTTAGTGTGCATTCATCTGA
TGGTGATTAAG

Initial ΔG = -48.30
```
CA  U       C         U             UC-|      UCCUU
  UA UCAUCA CAGAUGAAU CACACUAACAUGGG   AGCAAUC     \
  AU AGUGGU GUCUACUUA GUGUGAUUGUACUC   UUGUUGG     U
GA  U       A         C             UCA^      UAUUC
```
>gly_27   Gm15:28664008:28664115:+

CAACCTTTGGTCAACTAACTTGAAGGACGAATTCTCTTAGAGGGGGGTTTGACCTATCTTGTTACCTTTATGTATTTGTCTTTTCTAGTTG
GGTTTGGCTCATCATTG

Initial ΔG = -27.70
```
CAACCUUU     --      U-            UCUCU      ---------|  GU
       GGUCAA  CUAACU  GAAGGACGAAU      UAGAGG          GGG  U
       UCGGUU  GGUUGA  UUUUCUGUUUA      AUUUCC          UCC  U
GUUACUAC     UG      UC            UGU--      AUUGUUCUA^  AG
```

>gly_28   Gm18:10413935:10414028:+

ATTTTTAGCTTTCAAACCCCCTATCTTGGATCACAGCCCCATTGCCTATCATGGGGCTTGGAGTGGGGCTTGATCCAAGATAGGAAGCTGG
AAA

Initial ΔG = -50.80
```
A|         UCAAACCC               C         ----   AU
 UUUUUAGCUU        CCUAUCUUGGAUCA AGCCCCAUU    GCCU  C
 AAAGGUCGAA        GGAUAGAACCUAGU UCGGGGUGA    CGGG  A
-^         --------               -         GGUU   GU
```
>gly_29   Gm14:19673165:19673347:-

TCTAAGACGGTTATTACAAAATAACCGCCTTAGAAAGTTTATAAAGAAAGACTTTCTAAGACAGTTGTTTGTAATACCCATCTTAGAAAGT
ATACCTTCTAAGACGGTCATTACAAAATAACCGTTTTAGAAAGCATGCTTTGCCAGACTTTCTAAGACGATTGTTTGTAATAACCGTCTTA
G

Initial ΔG = -81.40
```
U|                 A   C  C            A-     AAAGA           A        -    AC  A        AGU
  CUAAGACGGUUAUUACAAA UAA CG CUUAGAAAGUUU  UAAAG      CUUUCUAAGAC GUUGUUU GUAAU  CC UCUUAGAA    A
  GAUUCUGCCAAUAAUGUUU GUU GC GAAUCUUUCAGA  GUUUC      GAAAGAUUUUG CAAUAAA CAUUA  GG AGAAUCUU    U
-^                 -   A  A            CC     GUAC-           C        A    CU  C        CCA
```

>gly_30   Gm08:8481417:8481647:+

CTAGCTAACATCAACGATTCCCGCTGTGTTCATAAGTGGGGAACGATCTAAACCGTATTCGATTCCCTTGCAAAATTCCAAAGCAGAGACT
GAAAATGTTCCGGCTGTATTTCCCAGAATAATAGCTAATTTTTCAGATGATTTTTTTGGGGGGATTTGTATATGTAAAAATATAAAATATG
AAAAGTTGCCTTCAATTCTGTGGACATGCCGGAGTGGTTATCGGGCATG

Initial ΔG = -56.70

```
CUA   AACAUC   G      C  U         .-AGU      -|   UCUAA
   GCU      AAC  AUUCC  GC  GUGUUCAUA     GGGGAA  CGA       A
   CGG      UUG  UGAGG  CG  UACAGGUGU     UCCCUU  GCU       C
GUA  GCUA--    G      C  -         \ ---      A^   UAUGC
                                                70-200
```

>gly_31   Gm13:41358339:41358461:+
GCCTGGCCCGACTGATCTGCATACATATCGTGTTGCCAAGCCGTCTCCATCAAAACAGGGACCAAGCGTGCCTGGAGACAGCAAGGCAGAA
CGATATGTACGCAGATCGGTTCGATCATGCAG

Initial ΔG = -60.80

```
--| C   CC   -          A          G     AA  C        UCAAAACA  G   CA
  GC UGG  CGA CUGAUCUGC UACAUAUCGU UUGCC  GC GUCUCCA         GG AC    \
  CG ACU  GCU GGCUAGACG AUGUAUAGCA GACGG  CG CAGAGGU         CC UG    A
GA^ U   A-   U          C          A     AA  A        --------  G   CG
```

>gly_32   Gm17:10900636:10900727:+
AAATTAGTTACAGGACCTCTCTTTTTTGGCACATCGCACACACTCGAGTTCCCAAATGATGCGCCAAAGAAGAGTTGTCCTGCAAGTGATT
C

Initial ΔG = -36.90

```
A     G  A      CU            A      CACAC--| U
 AAUUA UU CAGGAC  CUCUUUUUUGGC CAUCG         AC C
 UUAGU AA GUCCUG  GAGAAGAAACCG GUAGU         UG G
C     G  C      UU            C      AAACCCU^A
```

>gly_33a   Gm12:32340039:32340153:-
ATTAATTGGTCAAACTAATCTTGATTGGGCTGAATTGTGTGAAGAATTGTTGGGAGTTAGACCACAAGAAGGTGAACTTCAAGGCAGTGTG
GTCAAATTAAGTTGGCTGGCTCAC

Initial ΔG = -28.90

```
AUUAA UG    AC    C       G     AA   U-  GAAGAA--|   U   GAG
     U  GUCAA  UAAU UUGAUUG GCUG  UUG  GU         UUGU GG    U
     G  CGGUU  AUUA AACUGGU UGAC  AAC  CA         AACA CC    U
CACUC GU    GA    -       G     GG   UU  AGUGGAAG^   -   AGA
```

>gly_33b   Gm06:19079272:19079386:+
ATTAATTGGCCAAACTAATCTTGATTGGGCTGAATTGTGTGAAGAATTGTTGGGAGTCAGACCACAGAAAGGTGAACTTCAAGGCAGTGTG
GTCAAATTAAGTTGGCTGACTCAC

Initial ΔG = -30.30

```
AUUAA UG    AC    C       G     AA   .-UGUGAAGAA|   U  GAG
     U  GCCAA  UAAU UUGAUUG GCUG  UUG             UUGU GG    U
     A  CGGUU  AUUA AACUGGU UGAC  AAC             GACA CC    C
CACUC GU    GA    -       G     GG   \ ---------^   -   AGA
```

>gly_34   Gm16:30111973:30112055:+
CGAAAACTTTCATCTTCAACTTGCTCAACGGTACACTCAAGTATAATCATATCGTTGAGCAAGTTGAAGATGAATGTTGTCGG

Initial ΔG = -43.80

```
-|  A   U                        CACUCAA
 CGA AAC UUCAUCUUCAACUUGCUCAACGGUA       G
 GCU UUG AAGUAGAAGUUGAACGAGUUGCUAU       U
G^  G   U                        ACUAAUA
```

>gly_35   Gm14:41954319:41954474:-
TAGTATTCATAGGCTCACATAAAGTTTATATATATTTACACGCATGATATTTTAGCTTATACACGCATGATACTTTAGCCAAGTCACACGT
GATATTAGCTCAAGTTAATATTAGCTTTAGGTATATATTTGTAAATTTTAGGCACACGTAAATTT

Initial ΔG = -29.50

```
UAGUAUUCAUAG   CACA           U    U    AC---  A        UU----    U   -|    CA    ACUUU
```

```
           GCU    UAAAGUUUAUA AUAU UAC     GC UGAUAUU      AGCU AUA CACG  UGAU      \
           CGG    AUUUUAAAUGU UAUA AUG     CG AUUAUAA      UCGA UAU GUGC  ACUG      A
UUUAAAUGCACA  ----           U    U    GAUUU  -        UUGAAC   U  A^   AC    AACCG
```

>gly_36   Gm09:33092118:33092255:+
GTCAGGCTTCTAAGACGGTGCTGACATCACGACTGTCTTAGAAAGCCAAGCTTCAAAGACGGTGCTAATGTCGACAACGTCTTAGAAAGTC
AACCTTCAAAGACGGTCGTGACGTCAGCATCGTCTTTAAAGCCTGGT
Initial ΔG = -71.40

```
G|        CU              A              A    AGCCAA      - A      G   CUAA
 UCAGGCUU  AAGACGGUGCUGAC UCACGACUGUCUU GAA         GCUU C AAGACG UG     U
 GGUCCGAA  UUCUGCUACGACUG AGUGCUGGCAGAA CUU         UGAA G UUCUGC AC     G
U^        AU              C              A    CCAAC-      A A      A   AGCU
```

>gly_37a   Gm10:4733496:4733599:-
GCATACATTGTGTGCCTGGCTCCCTGTATGCCATTTGTAGACCCCATCACAATGGTGATGGCCTTATCAAATGGCGTATGAGGAGTCATGC
ATGCTGTGTTTTG
Initial ΔG = -52.20

```
 GCAU|    U       C        CU              UAGACC      A
     ACAU GUGUGC UGGCUCC  GUAUGCCAUUUG      CCAUCAC A
     UGUG CGUACG ACUGAGG  UAUGCGGUAAAC      GGUAGUG U
GUUU^    U       U        AG              UAUUCC      G
```

>gly_37b   Gm13:23749975:23750082:-
ATAAGCATACTTTGTGTGCCTGGCTCCCTGTATGCCATTTGTAGACCCCATTACAAAGGTGATGGCCTTAGCAAATGGCGTATGAGGAGTC
ATGCATGCTGTGTTTTG
Initial ΔG = -52.30

```
AU|     CUUU       C        CU                A  CC        A
  AAGCAUA    GUGUGC UGGCUCC  GUAUGCCAUUUGU GA  CCAUUAC A
  UUUGUGU    CGUACG ACUGAGG  UAUGCGGUAAACG UU  GGUAGUG A
GU^     ----       U        AG                A  CC        G
```

> gly_38   Gm18:34200523:34200636:+
CTATATATAATATGGATAACATGCAGAAGCTTTGTACTACGTTCTACAAGAATAAAAGCTTTTTATTCTCTAAATGTAGTAC
AAAGCTTCTATATGTTATCCATGTTAGTGTGC
Initial ΔG = -54.70

```
C|    A                 C                       CUACA         G
  UAUAU UAAUAUGGAUAACAUG AGAAGCUUUGUACUACGUU     AGAAUAAAA \
  GUGUG AUUGUACCUAUUGUAU UCUUCGAAACAUGAUGUAA     UCUUAUUUU C
C^    -                 A                       AUC--         U
```

>gly_39   Gm08:4270886:4271063:-
AAGAAAAATATCTTGGGACAGAGGTATCAATACTAAATTTTTTATTGAATTCCATCAAATAACAATGACAAATTTTCCTTAT
AACTCTTTGAATGACTTAACACAATTTATCCTTGTTATTTGATGGAGTTTAATAAAAAATTTAGTTTTGATATCACTGTCCA
AGATATTTTTCTTA
Initial ΔG = -79.40

```
-|                  G    A         U                                   U  C     UUCCUUA    C  UUU
  AAGAAAAAUAUCUUGG ACAG GGUAUCAA ACUAAAUUUUUUAUUGAAUUCCAUCAAAUAACAA GA AAAUU       UAA UC     \
  UUCUUUUUAUAGAACC UGUC CUAUAGUU UGAUUUAAAAAAUAAUUUGAGGUAGUUUAUUGUU CU UUUAA       AUU AG    G
A^                -    A         U                                   C  A    CACA---    C  UAA
```

> gly_40a   Gm02:9877923:9878114:-
GTTCTTTTTGTTCAATTTGATTTTTTAATATTTAAAATTAATTCATTTTTTTAAGAATGTGTATTTTATGATGATTTAAAATCATTTGATA
TTAATTTTGAATTGCCACAAAATTTAATTTCTTATAATTTAGACTCAAAAATAAAATTGAATCAATTTAAAAAATTAGAAGATCAAATTGA
ATCTATGAAA
Initial ΔG = -39.70

```
G    UUUUU                      A   A      A     U    UUAAGAAU  G  UU      U  .-U|   A    A
 UUC       GUUCAAUUUGAUUUUUUAAU UUU  AAAUU AUUCA UUUU         GU UA  UUAUGA GA    UUAA AUC U
 AAG       UAAGUUAAACUAGAAGAUUA AAA  UUUAA UAAGU AAAA         CA AU  AAUAUU CU    AAUU UAG U
A    UAUC-                      A   A      C     U    UAAAAACU  G  UU      -  \ -^   A    U
                                                                              90-120
```

>gly_40b   Gm11:36587596:36587859:+

TTTTTTAGGTTCAATTTGGTTATCTAGTTTTCTTAGACTGAATTTGTTTTTTTTTTTAATTTTAAAAATTGATTTAATTTGGTCATACAAT
TTAAATTAGATCAACAATAATAAGAAATAACACTTTCTCTTGAAATTTAAAATCATCACCGAGTGAGTGATTTTAAACCGTCTATACATTT
TCTAACATGTCATTTTTTTTTTTATTGACAAGATCAAATTAAAATAATTTAAAAAATTAGAAGATCAAATTGAA

Initial ΔG = -54.10

```
UUUUUUUAGG            A         .-CUUA   UG     U-|   UU     UUA
          UUCAAUUUGGUU UCUAGUUUU      GAC   AAUU   GUU  UUUUU   A
          AAGUUAAACUAG AGAUUAAAA      CUG   UUAA   UAG  AAAAA   U
---------             A         \ ----   GU     UU^   UU    UUU
                               90-230
```

> gly_40c   Gm18:52073025:52073212:+

TAATTTTTTGTTCGATTTGGTCATTTAATTTTTAAAATAATTTTTTTTTATAAAATATATATTTTGTGATGATTTAAAATCACTTAATGAG
TTTTGAGCTATCACAAAGGGTGATTTTTTACCATTTAAAATGAAAAAATAAATTGAATCAATTTAAAAAATTAGAAGATCAAATTGAAAAA
AAAATT

Initial ΔG = -47.00

```
U        G            A          .-AAAAUAAUU         .-AAAAUAUAUAU        A       -|  CU
 AAUUUUUU UUCGAUUUGGUC UUUAAUUUUU           UUUUUUUAU             UUUGUGAUG UUUAAAA  UCA   \
 UUAAAAAA AAGUUAAACUAG AGAUUAAAAA           AAAAAAGUA             AAACACUAU GAGUUUU  AGU   U
-        A            A          \ ---------         \ -----------        C       G^  AA
```

> gly_41a   Gm03:41268407:41268513:-

TTAGCATACTTGGCGTGCCTGGCTCCCTGTATGCCATTTGCAGAGCCCATCAGGACAGTGATGGCCTTAACGAATGGCGTGCGAGGAGCCA
TGCATGCTGTGTGTTA

Initial ΔG = -63.80

```
U|        UU       C       C             CA   C      GG
  UAGCAUAC  GGCGUGC UGGCUCC UGUAUGCCAUUUG  GAG CCAUCA  A
  AUUGUGUG  UCGUACG ACCGAGG GCGUGCGGUAAGC  UUC GGUAGU  C
-^        --       U       A             AA   C      GA
```

> gly_41b   Gm19:43795944:43796051:-

ATTAGCATACTTGGCGTGCCTGGCTCCCTGTATGCCATTTGCAGAGCCCATCAGGACAGTGATGGCCTTAACGAATGGCGTGCGAGGAGCC
ATGCATGTTGTGCGTTA

Initial ΔG = -58.50

```
AUUA|     CUUG     C       C             CA   C      GG
     GCAUA   GCGUGC UGGCUCC UGUAUGCCAUUUG  GAG CCAUCA  A
     CGUGU   UGUACG ACCGAGG GCGUGCGGUAAGC  UUC GGUAGU  C
AUUG^     ----     U       A             AA   C      GA
```

> gly_42   Gm14:13037548:13037788:+

GTATTTGTTGAGTTGATGATAATAAATGAACCTTAAACTAGATAGTGACCTTTGTATGCATGAAGGGATGATTTGGTGAAGTCAACCCCAA
CACTTTTATTCATTGTTGTTTATGTTTTTTTTTTACCACATTCCAAGTATTTGTAAAAATTACCAAAAACAGTTTATTGTTCTTTTACTCT
TTAATTTTTTTGTTTATTTTAGTTCTTGGCATTTACATTATCGCAATTCAATTAAAACC

Initial ΔG = -41.30

```
-  A  U        A     --      AA  UUA      .-UA   CC   U--|    AUGAA    AUU      UC--   CC---    CUUUU
 GU UU GUUGAGUUG UGAUAA  UAAAUG  CC   AACUAGA   GUGA  UUUG   AUGC     GGGAUG  UGGUGAAG    AAC    CAACA    \
 CA AA UAACUUAAC GCUAUU  AUUUAC  GG   UUGAUUU   CAUU  AAAU   UAUG     CCUUAC  ACCAUUUU    UUG    GUUGU    A
C  A  U        -     AC      --  UUC      \ --   AA   GUU^   AA---    ---      UUUU   UAUUU    UACUU
                                                  160-170
```

```
> gly_43   Gm15:16622255:16622354:+
AAGGATATATTTTTAAGACGGTTGTTAGCTAAAAATCATCTTAGAGGAGTATATTTTTTAAGATGATTTATGTAAGAACTGTCTTAAAATC
ATTTTTTTA
Initial ΔG = -33.30
-|     U  AU              G    G UAA                 GU
  AAGGA AU  UUUUAAGACGGUU UUA C    AAAUCAUCUUAGAGGA  \
  UUUUU UA  AAAAUUCUGUCAA AAU G    UUUAGUAGAAUUUUUU  A
A^     U  CU              G    - UA-                 AU
> gly_44a   Gm02:38406240:38406364:+
ATGCATGTTCCTCCTCTACCAGCTTAATATCTTCCGTTTCACCTTTGCTTTTGTAAACTACACTGTAATTTACAAAAAAAAAGTTGAAAGG
AAAGATATTGAGCTGGTAGATGAGGAAGAAGCAC
Initial ΔG = -60.10
A   AUG      C                 -|   G     C    GC          C   A
 UGC   UUCCUC UCUACCAGCUUAAUAUC UUCC UUUCA CUUU  UUUUGUAAA UAC \
 ACG   AAGGAG AGAUGGUCGAGUUAUAG AAGG AAAGU GAAA  AAAACAUUU AUG C
C   AAG      U                 A^   -     U    AA          A   U
>gly_44b   Gm04:36054334:36054457:-
CGGCTCTTCCTCCTCTACCAACTCAATATCTTTCGTTTCACCTTTGCTTTTGTAAACTACACTGTAATTTACAAAACAAAAGTTGAAAGGA
AAGATATTGAGCTGGTAGATGAGGAAGAAGCAC
Initial ΔG = -63.80
CG  -|        C       A             G     C    GC         C    A
  GC UCUUCCUC UCUACCA CUCAAUAUCUUUC UUUCA CUUU  UUUUGUAAA UAC \
  CG AGAAGGAG AGAUGGU GAGUUAUAGAAAG AAAGU GAAA  AAAACAUUU AUG C
CA  A^        U       C             G     U    AC         A    U
> gly_44c   Gm07:21834157:21834282:-
TGTGTCTGTTCCTCCTCTACCAGCTCAATATCTTTCGTTTCACCTTTGCTTTTGTAAACTACACTGTAATTTACAAAACAAAAGTTGAAAG
GAAAGATATTGAGCTGGTAGATGAGGAAGAAGCAC
Initial ΔG = -66.20
U|  U  G-     C                       G     C    GC          C   A
 GUG CU  UUCCUC UCUACCAGCUCAAUAUCUUUC UUUCA CUUU  UUUUGUAAA UAC \
 CAC GA  AAGGAG AGAUGGUCGAGUUAUAGAAAG AAAGU GAAA  AAAACAUUU AUG C
-^  -  AG     U                       G     U    AC          A   U
> gly_44d   Gm10:27337135:27337260:+
CGTGGCTCTTCCTCCTCTACCAGCTCAATATCTTTCCTTTCACCTTTGCTTTTGTAAACTACACTGTAATTTACAAAACAAAAGTTGAAAG
GAAAGATATTGAGCTGGTAGATGAGGAAGAACCAC
Initial ΔG = -78.20
C|   C        C                              C    GC          C   A
 GUGG UCUUCCUC UCUACCAGCUCAAUAUCUUUCCUUUCA CUUU  UUUUGUAAA UAC \
 CACC AGAAGGAG AGAUGGUCGAGUUAUAGAAAGGAAAGU GAAA  AAAACAUUU AUG C
-^   A        U                              U    AC          A   U
>gly_44e   Gm14:12615238:12615363:+
CGTGCCTGTTCCTCCTCTACCAGCTCAATATCTTTATTTTCACTTTTGCTTTTGTAAACTACATTGTAATTTACAAAACAAAAGTTGAAAG
GAAAGATATTGAGCTGGTAGATGAGGAAGAAGCAC
Initial ΔG = -66.60
C    CUG     C                    A     -|      C          C   A
 GUGC   UUCCUC UCUACCAGCUCAAUAUCUUU UUUUC ACUUUUG UUUUGUAAA UAC \
 CACG   AAGGAG AGAUGGUCGAGUUAUAGAAA GAAAG UGAAAAC AAAACAUUU AUG U
-    AAG     U                    G     U^      -          A   U
>gly_44f   Gm18:15474680:15474805:+
CGTGCCTGTTCCTCCTCTACCAGCTCAATATCTTTCGTTTCACCTTTGCTTTTGTAAACTACACTGTAATTTACAAAACAAAAGTTGAAAG
GAAAGATATTGAGCTGGTAGATGAGGAAGAAGCAC
Initial ΔG = -67.80
C|   CUG     C                      G     C    GC          C   A
 GUGC   UUCCUC UCUACCAGCUCAAUAUCUUUC UUUCA CUUU  UUUUGUAAA UAC \
 CACG   AAGGAG AGAUGGUCGAGUUAUAGAAAG AAAGU GAAA  AAAACAUUU AUG C
-^   AAG     U                      G     U    AC          A   U
>gly_44g   Gm18:12719394:12719518:-
ATGCATGTTCCTCCTCTACCAGCTCAATATCTTTCGTTTCACCTTTGCTTTTGTAAACTACACTGTAATTTACAAAACAAAAGTTGAAAGG
AAAGATATTGAGCTGGTAGATGAGGAAGAAGCAC
Initial ΔG = -65.60
```

```
A|  AUG      C                    G     C    GC         C   A
 UGC   UUCCUC UCUACCAGCUCAAUAUCUUUC UUUCA CUUU  UUUUGUAAA UAC \
 ACG   AAGGAG AGAUGGUCGAGUUAUAGAAAG AAAGU GAAA  AAAACAUUU AUG C
C^  AAG      U                    G     U    AC         A   U
>gly_44h  Gm19:7602747:7602871:+
GTGCCTGTTCCTCCTCTACCAGCTCAATATCTTTCGTTTCACCATTGCTTTTGTAAACTACACTGTAATTTATAAAACAAAAGTTGAAAGG
AAAGATATTGAGCTGGTAGATGAGGAAGAAGCAA
Initial ΔG = -61.40
G   CUG      C                    G     -| CA   C          C   A
 UGC   UUCCUC UCUACCAGCUCAAUAUCUUUC UUUC AC  UUG UUUUGUAAA UAC \
 ACG   AAGGAG AGAUGGUCGAGUUAUAGAAAG AAAG UG  AAC AAAAUAUUU AUG C
A   AAG      U                    G     U^ AA   -          A   U
>gly_45   Gm05:14712477:14712591:-
TAATGAACAAGTTTCTTAACTATTTGTCCTTAAGATTTTACTATTTCTTTTTGCTAATATGTAAATATAAATGGTGTAAGGGCTAAAGGAC
AAATAGTTAAGAAAGTTGTTCATT
Initial ΔG = -50.20
U          G                    A  A-    -|       CUU     UA
 AAUGAACAA UUUCUUAACUAUUUGUCCUU AG  UUU  UACUAUUU   UUUGC  A
 UUACUUGUU AAAGAAUUGAUAAACAGGAA UC  GAA  GUGGUAAA   AAAUG  U
-          G                    A  GG    U^       UAU     UA
>gly_46   Gm16:2717409:2717580:+
AATGATAACAAGGTAAAATGAATTGAGTTTCTTTGTGAGTTAAAATCAACTTATGCACCTCACTTTTAGTTTTTGAAGAAGTTAAATGAAA
GAGTTTCTATAGAAGTTAAGTGTGTAAATTGATTTTAAGTTATACAAAAAAATCCAATTCATTTTACCCTTAAAATTATTT
Initial ΔG = -46.20
-      AACAA-              AG    C      --| G          C        CUC       GUUUUU    AAG-  AA
 AAUGAU      GGUAAAAUGAAUUG  UUU UUUGU  GA UUAAAAUCAA UUAUGCAC   ACUUUUA      GAAG    UU  \
 UUAUUA      CCAUUUUACUUAAC  AAA AAACA  UU AAUUUUAGUU AAUGUGUG   UGAAGAU      CUUU    AA  A
U      AAAUUC              CU    A      UA^ G          A        AAU       AU----    GAGA  GU
>gly_47   Gm16:28407538:28407722:+
GAGAGGAAGGAGGCTGATAGTTCAACAAGGCAAGCTTTTCTTCATTTATATTGTGAAAAGTCTGTCTAGTATCCACCCTACCATCTGAATT
CAGATTTCTTGAGAAAGAATGTCCCTTGCCATGAATGCCTCTAGAAATTAGGTGAGAAGCTTGCCTTGTCGAACTATCAGCCTCCTTCCTC
TCT
Initial ΔG = -88.60
-                          A                .-U|   UUA
 GAGAGGAAGGAGGCUGAUAGUUC ACAAGGCAAGCUUUUC   UCAU   \
 CUCUCCUUCCUCCGACUAUCAAG UGUUCCGUUCGAAGAG   AGUG   U
U                          C                \ -^   UUA
                                            60-140

> miR4416d  Gm02:30498955:30499126:-
TGTGGTCACTTCTGCTCTGGGTGAGAGAAACACGTATTGAAGAAGGTTCATGGCTTAGACAATAAGTTGCTACTTTGCTGAACTCACCATT
TTATGTGAAGAAGTGGCTATGCTAATTGTTTGATTCTGTACACGTACGATACGGGTCGCTCTCACCTGGAGTAGGGCTGCC
Initial ΔG = -65.30
U|UG   ACUU      UG         AA  A         AAGAAG  U   U GC         A    U--       GCUGAAC    CAU
 G  GUC    CUGCUC  GGUGAGAG  AC  CGUAUUG       GU  CA  G  UUAGACAAU AGU   GCUACUUU       UCAC    U
 C  CGG    GAUGAG  CCACUCUC  UG  GCAUAGC       CA  GU  C  AGUUUGUUA UCG   CGGUGAAG       AGUG    U
C^GU   ----      GU         GC  G         AUGCA-  U   - UU         A    UAU       A------    UAU
> miR399i   Gm08:9112036:9112142:-
AGGTGCATTACTGGGCAATGACTTATTTGGCAGTTGAATTGACCATTCAGGCCATGACCAGAGAAGCTATGTTTACCGCCAAAGAAGATTT
GCCCGGCAATTCATCC
Initial ΔG = -31.40
A|   C    A        UGA   A        A  U     UGACCA    A--   CC
 GGUG AUU CUGGGCAA   CUU UUUGGC GU GAAU        UUC     GG   A
 CUAC UAA GGCCCGUU   GAA AAACCG CA UUUG        AAG     CC   U
C^   U    C        UA-   G        C  -     UAUCG-    AGA   AG
> miR399j    Gm09:34174615:34174748:+
GTAAGCAAATCATTCATAGGGCATGTCTCTTTTGGCAGGTAATGGTGATTACTTTGGATTAATTCAGAGCTACCAAAATATGCCTCATAAA
TAGTAATCAACTTGCCAAAGGAGAGCTGCCCTGTGAGTGCTTG
Initial ΔG = -54.80
G       AAUCA            UG                .-AA|      AUUA        U
```

```
 UAAGCA     UUCAUAGGGCA  UCUCUUUUGGCAGGU     UGGUG    CUUUGGA U
 GUUCGU     GAGUGUCCCGU  AGAGGAAACCGUUCA     ACCAU    GAGACUU A
-      -----           CG               \ --^     C---       A
> miR399k  Gm16:35606655:35606782:+
GCAAATCATTCATAGGGCATGTCTCTTTTGGCAGGTAATGGTGATTACTTTGGATTAATTTAGAGCTACCAAAATATGTCTTATATAGTAA
TCAACTTGCCAAAGGAGAGCTGCCCTGTGAGTGTTTG
Initial ΔG = -55.80
G|    U              UG               AAUGG        UU---    UA    AGAG
  CAAA CAUUCAUAGGGCA   UCUCUUUUGGCAGGU     UGAUUACU     GGAU  AUUU    C
  GUUU GUGAGUGUCCCGU   AGAGGAAACCGUUCA     ACUAAUGA     UCUG  UAAA    U
-^    -              CG                -----       UAUAU    UA    ACCA

> miR4416c  Gm19:40699070:40699221:-
TCCTATGGTCATCTTTGATCTGGGTGAGAGAAACGCGTATCGATGGATTGGGTTCAGTTCTGGTCTCACACGGTTTGTTCTAACAATTTGT
ACTGACTGTGTTTTGATCGATACGGGTCGCTCTCACCTAGGCCAGAGTTGCCCAAACTTTG
Initial ΔG = -67.50
UCCUA--   U  U    AU           AA  G         .-G|  UG  UU
       UGG CA CUUUG CUGGGUGAGAG  AC CGUAUCGAU   GAU  GG  C
       ACC GU GAGAC GAUCCACUCUC  UG GCAUAGCUA   CUG  CU  A
GUUUCAA   C  U    CG           GC  G         \ -^  GU  UG
                                              70~100
>miR408e  Gm19:47163685:47163821:-
CAGGGCAAAGGCTGGGAACAGGCAGAGCACGAATGGAGCTATCAACAGAAAATGGAGAAGTGAGAAATGAGAGGAGAGAAAG
AGAGAGATCTGTTAGGGCTACGCTCATGCACTGCCTCTTCCCTGGCTCTGTCTCT
Initial ΔG = -42.69
CA|    AA    -     C      A   C  A  G    AUC      AAAUGGAGAAGUGAGAAAU
  GGGCA  GGCU GGGAA AGGCAG GCA GA UG AGCU   AACAGA                   G
  UCUGU  UCGG CCCUU UCCGUC CGU CU GC UCGG   UUGUCU                   A
UC^    C-    U     C      A   A  C  A    GA-      AGAGAGAGAAAGAGAGGAG

> miR156aa    Gm18:61442586:61442692:-
GGTGATGCTGCTGACAGAAGATAGAGAGCACTGATGATGAAATGCATGAAAGGGAATGGCATCTCACTCCTTTGTGCTCTCTAGTCTTCTG
TCATCATCCTTCTCCC
Initial ΔG = -43.90
-  U  UGC-  C          -          U  U  -   A   -|  GAA
 GG GA    UG UGACAGAAGA UAGAGAGCAC GA GA UGA AUG  CAU  A
 CC CU    AC ACUGUCUUCU AUCUCUCGUG UU CU ACU UAC  GUA  G
C  U  UCCU  U          G          U  C  C   C   G^  AGG
> miR482f  Gm18:61452908:61452997:-
AGAATTTGTGGGAATGGGCTGATTGGGAAGTAATGAGATTGAGCAATACATTTAATTTCTTCCCAATTCCGCCCATTCCTATGATTTCTG
Initial ΔG = -41.00
-    U UG          U-|         GUAAU         CAA
 AGAA U UGGGAAUGGGC   GAUUGGGAA     GAGAUUGAG    \
 UCUU A AUCCUUACCCG   UUAACCCUU     CUUUAAUUU    U
G    U GU          CC^         -----         ACA
> miR164l  Gm07:3508921:3509000:+
CGAACCACGCTGGAGAAGCAGGGCACATGCTTGATTTATGTAAGTTTTGAGCTTGTGTCCTACTTCTCCAGCATGGTTTC
Initial ΔG = -45.80
C|      C          C        U   UG UUUAU
  GAACCA GCUGGAGAAG AGGGCACA GCU  A     G
  UUUGGU CGACCUCUUC UCCUGUGU CGA  U     U
C^      A          A        U   GU UUGAA

Initial ΔG = -55.20
----| UUU  UAAUUUGU  A          -            U     CUUUCC         U
    GG   GU       GG  GGUGACAGA AGAGAGUGAGCAC UGGCU      UAGCAUGGA \
    CC   CA       CC  CCACUGUCU UCUCUUACUCGUG GUCGA      AUCGUGCUU C
CUCU^  CU-  UUUUCCU- A          A            U     AA----         U
> miR396l  Gm17:35366529:35366672:-
```

```
GAATGGTCTTTTTCGTGATCTTCCACAGCTTTCTTGAACTGTGTTGTGAGGCTTCTCTCCAATGAAGGTTTATACCCTATGCAAAAGAAAT
TCTATGAGCACAATTCAAGATAGCTGTGGAAAATCACTGAGATGATCTGGTTC
Initial ΔG = -53.30
    --     U    C     C          U       C      |       G    UCU
GAAU   GGUC UUUU GUGAU UUCCACAGCU UCUUGAA UGUGU--UGUGAG CUUC    C
CUUG   CUAG AGAG CACUA AAGGUGUCGA AGAACUU ACACG  AUAUUU GAAG    C
    GU     U    U     A          U       A     \ ^      G    UAA
                                                 76-96
```

```
>miR156ab   Gm06:4699132:4699253:-
TTCTTGGGACATAGAAATTGACAGAAGAGAGTGAGCACACAGAGGCACTTGGTATAGTATATATACTATTGCTTTTGCGTGCTCACTTCTC
TTTCTGTCAACTTCCAGTGCTGGAAAATTCC
Initial ΔG = -56.10
UUCUU    .-CAUAGAAA      -     -|         A       CU       GU
     GGGA          UUGACAG AAGAG AGUGAGCAC CAGAGGCA  UGGUAUA  \
     CCUU          AACUGUC UUCUC UCACUCGUG GUUUUCGU  AUCAUAU  A
-----    \ --------      U     U^         C       U-       AU
>miR156ac   Gm04:4990842:4990962:-
TCTTTGGAACTTAGAAATTGACAGAAGAGAGTGAGCACACAGAAGCACTTGGTATAGTTATATACTGTTGCTTTTGCGTGCTCACTTCTCT
TTCTGTCAACTTCCAGTGCTGGAAAATTCC
Initial ΔG = -55.60
UCUUU    .-CUUAGAAA      -     -|         A       CU       G
    GGAA           UUGACAG AAGAG AGUGAGCAC CAGAAGCA  UGGUAUA U
    CCUU           AACUGUC UUCUC UCACUCGUG GUUUUCGU  GUCAUAU U
-----    \ --------      U     U^         C       U-       A
>miR156ad   Gm17:38431866:38431973:-
AAGGGAGGTGACAGAAGAGAGTGAGCACACATAGTACTTTCTTGAATGATATAAGGTTTCATGCTTGAAGTTATGCGTGCTTACTCTCTAT
CTGTCACCCATCACCAT
Initial ΔG = -49.80
AA  -  --         -|            A    GU     CUUGA    UAUA
  GG GA  GGUGACAGA AGAGAGUGAGCAC CAUA  ACUUU     AUGA     \
  CC CU  CCACUGUCU UCUCUCAUUCGUG GUAU  UGAAG     UACU     A
UA  A  AC         A^            C    --     UUCG-    UUGG
>miR156ae   Gm14:9431590:9431718:+
AATGAAGGGTAAGAGAGGTGACAGAAGAGAGTGAGCACACATGGTATTTTCTTGGATGATATAAGATTTCATGCTTGAAACTATGCGTGCT
CATTCTCTATCTGTCACCCATCATCATTTCTTTCTTGT
Initial ΔG = -54.70
AAU-      UAA  -  --         -|            A      AUU   UU   -    UAUA
   GAAGGG   GA GA  GGUGACAGA AGAGAGUGAGCAC CAUGGU   UUC  GG AUGA     \
   CUUUCU   CU CU  CCACUGUCU UCUCUUACUCGUG GUAUCA   AAG  UC UACU     A
UGUU      UUA  A  AC         A^            C      ---   U-   G    UUAG
>miR156af   Gm04:4257059:4257160:+
AAGGGAGGTGACAGAAGAGAGTGAGCACACATAATACTTTCTTGTAAGATAAAGATTCATGCTTGAAGTTATGTGTGCTCACTCTCTATCT
GTCACCCCACC
Initial ΔG = -58.60
AAG| A          -                    ACU   UU   A  UAA
   GG GGUGACAGA AGAGAGUGAGCACACAUAAU   UUC  GUA GA   \
   CC CCACUGUCU UCUCUCACUCGUGUGUAUUG   AAG  CGU CU   A
CCA^ -          A                    ---   UU   A  UAG
```

```
>miR156ag   Gm06:4013572:4013673:+
AAGGGAGATGACAGAAGAGAGTGAGCACACATGGTATTTTCTTGCACGATGACGATTCATGCTTGAAGCTATGTGTGCTTACTCTCTATCT
GTCATCCCATC
Initial ΔG = -58.40
AAG|  A         -                    AUU    UU    C  UGA
   GG GAUGACAGA AGAGAGUGAGCACACAUGGU    UUC  GCA GA     \
   CC CUACUGUCU UCUCUCAUUCGUGUGUAUCG    AAG  CGU CU     C
CUA^  -         A                    ---    UU    A  UAG
>miR397c   Gm08:4639046:4639152:-
```

GAGAAACATCATTGAGTGCAGCGTTGATGAAGTTTCACTCTCATCTCAGGTAGATGCTTAATTTATAGTGTTATTGTCATCGACACTGCAC
TCAATCATGTTTTTCT

Initial ΔG = -45.70

```
-         C           C        -   UU     CU--------|     CA
 GAGAAACAU AUUGAGUGCAG GUUGAUGA AGU  CACU           CAUCU    \
 CUUUUUGUA UAACUCACGUC CAGCUACU UUA  GUGA           GUAGA    G
U         C           A        G   UU     UAUUUAAUUC^     UG
```

>miR1513e   Gm17:1401437:1401518:-

GATATGAGAGAAAGCCATGACTTACACACGCATTGAAATCTAAGTTAAATATGTGTGTAAGTCATGGCATTATCTCATATCT

Initial ΔG = -42.20

```
-|         G  A                 C    GAAAUC
  GAUAUGAGA AA GCCAUGACUUACACACG AUU        \
  CUAUACUCU UU CGGUACUGAAUGUGUGU UAA        U
U^         A  A                 A    AUUGAA
```

>miR167k   Gm02:307523:307611:-

CACTACTGGTTGAAGCTGCCAGCCTGATCTTAACTTTTCTCTTTTATTAGGAAAAGATCAGATCATGTGGTAGCCTTCACCTAGTTGTT

Initial ΔG = -34.30

```
C  U     U     -|       G C      UAA      U  UU
 AC ACUGG UGAA  GCUGCCA C UGAUCU    CUUUUC CU   U
 UG UGAUC ACUU  CGAUGGU G ACUAGA    GAAAAG GA   A
U  U     C     C^       - U      CUA      -  UU
```

miR167i   Gm02:307523:307611:-

CACTACTGGTTGAAGCTGCCAGCCTGATCTTAACTTTTCTCTTTTATTAGGAAAAGATCAGATCATGTGGTAGCCTTCACCTAGTTGTT

Initial ΔG = -34.30

```
C  U     U     -|      G C      UAA      U  UU
 AC ACUGG UGAA GCUGCCA C UGAUCU   CUUUUC CU  U
 UG UGAUC ACUU CGAUGGU G ACUAGA   GAAAAG GA  A
U  U     C     C^      - U      CUA      -  UU
```

>miR2111g   Gm01:31787453:31787538:+

CCGGATTAGGTAATCTGCATCCTGAGGTTTAGGACAATATCCTTTGTTTTGTTTCTAGTCCTTGGGATGCAGATTACGTCTTCCTT

Initial ΔG = -39.80

```
CC   UUAG             UG   U     -|     UAUCC
  GGA    GUAAUCUGCAUCC  AGG UUAG GACAA       \
  CCU    CAUUAGACGUAGG  UCC GAUC UUGUU       U
UU   UCUG             GU   U     U^     UUGUU
```

>miR2111h   Gm07:16402351:16402433:-

TGGGAAAAGCTAATCTGCATCCTGAGGTTTAGAGCAATGTTTTTGTTGCATCTAGTCCTTGGGATGCAGATTATCTCTTCCTT

Initial ΔG = -45.60

```
U      A  C            UG    U      -|    GU
 GGGAA AG UAAUCUGCAUCC  AGG UUAGA GCAAU  U
 UCCUU UC AUUAGACGUAGG  UCC GAUCU CGUUG  U
U      C  U            GU    U      A^    UU
```

>miR2111i   Gm01:31787453:31787538:+

CCGGATTAGGTAATCTGCATCCTGAGGTTTAGGACAATATCCTTTGTTTTGTTTCTAGTCCTTGGGATGCAGATTACGTCTTCCTT

Initial ΔG = -39.80

```
CC   UUAG             UG   U     -|    UAUCC
  GGA    GUAAUCUGCAUCC  AGG UUAG GACAA     \
  CCU    CAUUAGACGUAGG  UCC GAUC UUGUU     U
UU   UCUG             GU   U     U^    UUGUU
```

>miR2111j   Gm18:49934381:49934486:-

AGTATTGGTTTAGGATCAGGTAATCTGCATCCTGAGGTTTAGAAACAATATGTTTATCGGGTCTAATCCTTGGGATGTAGATTACCACGTC
CTTATGTTATGACCA

Initial ΔG = -44.00

```
AGUAU     U-------|     CA              UG    U     AACA   U
     UGGUU             AGGAU  GGUAAUCUGCAUCC  AGG UUAGA    AUA G
     ACCAG             UCCUG  CCAUUAGAUGUAGG  UCC AAUCU    UAU U
-----     UAUUGUAU^     CA              GU    U     GGGC   U
```

>miR399e   Gm05:34951412:34951529:+

TGTGCTGTTGAGGAATGATAGAGCAAATCTCCAGTGGCAGAGAGAAGTAGAGGGCTTGAACATGCATGCTTCTGGTTTGCCAAAGGAGATT
TGCCCTGCCATTTTTTAACAGTTTCAG

```
Initial ΔG = -54.40
-  U-               A   A          AG       G-|      GAGG   UUG
 UG  GCUGUUGAGGAAUG UAG GCAAAUCUCC  UGGCAGA  AGAAGUA     GC    A
 AC  UGACAAUUUUUUAC GUC CGUUUAGAGG  ACCGUUU  UCUUCGU     CG    A
G  UU               C   C          AA       GG^      A---   UAC
>miR399f   Gm08:9114311:9114428:+
TGTGCTGTTGAGGAATGATAGAGCAAATCTCCATTGGCAGTGGGAAGTAGAGGGCTTGAACATGCATGCTTCTGGTTTGCCAAAGGAGATT
TGCCCTGCCATTTTTTAACAGTTTCTG
Initial ΔG = -53.80
UGU--               A   A          A       UG-|      GAGG   UUG
     GCUGUUGAGGAAUG UAG GCAAAUCUCC UUGGCAG   GGAAGUA     GC    A
     UGACAAUUUUUUAC GUC CGUUUAGAGG AACCGUU   UCUUCGU     CG    A
GUCUU               C   C          A       UGG^      A---   UAC
>miR398d   Gm08:14299237:14299369:-
GTAGGTAGAGGGATTCTACAGGGTCGTCCTGAGATCACATGAAGTAGAATATAAGCAGCCATATTTGCTTCTTGTCTCGTGTTCTCAGCTC
ATGTGTTCTCAGGTCGCCCCTGCTGGACTATTCTCTACCAAC
Initial ΔG = -60.80
GUA         AU-    -     U  U       U      A   --        --------|     CC
   GGUAGAGGG   UCUA CAGGG CG CCUGAGA CACAUG AGU  AGAAUAU          AAGCAG   A
   CCAUCUCUU   AGGU GUCCC GC GGACUCU GUGUAC UCG  UCUUGUG          UUCGUU   U
CAA         AUC    C     C  U       U      -   AC        CUCUGUUC^     UA

>miR399n   Gm05:34951412:34951528:+
TGTGCTGTTGAGGAATGATAGAGCAAATCTCCAGTGGCAGAGAGAAGTAGAGGGCTTGAACATGCATGCTTCTGGTTTGCCA
AAGGAGATTTGCCCTGCCATTTTTTAACAGTTTCA
Initial ΔG = -53.80
UGU-               A   A          AG       G-|     GAGG  UUG
    GCUGUUGAGGAAUG UAG GCAAAUCUCC  UGGCAGA  AGAAGUA    GC   A
    UGACAAUUUUUUAC GUC CGUUUAGAGG  ACCGUUU  UCUUCGU    CG   A
ACUU               C   C          AA       GG^     A---  UAC
> miR399o   Gm08:9114319:9114417:+
TGAGGAATGATAGAGCAAATCTCCATTGGCAGTGGGAAGTAGAGGGCTTGAACATGCATGCTTCTGGTTTGCCAAAGGAGAT
TTGCCCTGCCATTTTTT
Initial ΔG = -42.90
U        A   A          A       UG-|      GAGG  UUG
 GAGGAAUG UAG GCAAAUCUCC UUGGCAG     GGAAGUA    GC    A
 UUUUUUAC GUC CGUUUAGAGG AACCGUU     UCUUCGU    CG    A
-        C   C          A       UGG^      A---  UAC
>miR1520s   Gm13:27429341:27429439:-
TGACTGTCATGTGTTATGTTCTGATTGGATGTTAACTCCATGAAAAATGAAATGCATTGTTATATTTATTGACCAATCAAAACATGACACA
TGATAGTC
Initial ΔG = -37.80
U|                    C       AUG      CUCCAUGAAA    AA
  GACUGUCAUGUGUUAUGUU UGAUUGG   UUAA             AAUG   \
  CUGAUAGUACACAGUACAA ACUAACC   AGUU             UUAC   A
-^                    A       ---      AUUUAUAUUG    GU

>miR4357b   Gm05:28486211:28486397:-
TCTATCTATGAACTGTACAAAACACACGACTCCTCAATTTTTCTCAAAATAATTTTATCTCGTTGTGCCTCAAAGTGATTAAATTCGTCGG
GTTTCCGCAGTGGTTCCCATCACAATACTCATCGCACATTAACTCATCACCCTTAAAGGGTCTTACAGTCGTGTGATTGTACGGTTCATAA
CTCAC
Initial ΔG = -51.30
UCUAUC              AA          .-CCUCAAUUUUUCUCAAAAUAAUUUUAUCUCGU     CUCAA     .-AUUAAAUUC|U    G
      UAUGAACUGUACAA  CACACGACU                                 UGUGC      AGUG              G CGG U
      AUACUUGGCAUGUU  GUGUGCUGA                                 ACACG      UCAU              C GCC U
CACUCA              A-          \ --------------------------------     CUAC-     \ ---------^-    U
                               UUAACUCAUC   UU
                                         ACCC  \
                                         UGGG  A
                               CAUUC-----   AA
```

```
>miR4357c   Gm16:25363104:25363290:-
TCTATCTATTAACTGTACAAAACACATGACTCCTCAATTATTCTCAAAATAATTTTAAGTCATCGCACCTCAAAGTGATTGAACTCGTCGG
GTTTCCACGGTGGATCCCATCACAATACTCGTCGCACACTAATTCGTCGTCCTTAAAGGGTTTTACAGTCGTGTGATTGTACGGTTCATAG
CTCGC
Initial ΔG = -44.10

UCUAU    U          AA          ------  .-C|        CU
     CUAU AACUGUACAA  CACAUGACU      CCU     AAUUAUU   \
     GAUA UUGGCAUGUU  GUGUGCUGA      GGG     UUAAUAA   C
CGCUC    C          A-          CAUUUU  \ -^         AA
                                       (60-140)
>miR166v   Gm19:36649678:36649997:-
AAGGAAAGGAGGGTGTCTGGAATGAAGTTTGATCCGAAATCATTGTCTCAGTTTCGTTCATTTTAACTTTGCTTGTTGTCAAAGAGAGGCA
GAAAGAGAGAGAGAGAGAGAGAGATTGGTCATGATCTGAGAAGGAAAGGGGGTGTTTGGAATGAGGTTTGATCCAAGATCATCACATCATATA
TATATCTTCTTCTTCAATTTCCCTTTCCTTCTTGATTTACTTACCTTCATACATATATTATTATTTTCATCACTTCATGCATGGGATGAGA
CTGATCTCGGACCAGGCTTCATTCCTCACACCTAACTTTCCTTGTAG
Initial ΔG = -112.80
----       GA      CU             A     A    .-UU|    AG     G    A    AAC       G  GU
   AAGGAAAG  GGGUGU  GGAAUGAAGUUUG UCCGA AUCA    GUCUC  UUUC UUC UUUU   UUUGCUU UU   C
   UUCCUUUC  UCCACA  CCUUACUUCGGAC AGGCU UAGU    UAGAG  AGAG GAG AGAG   AGACGGA AG   A
GAUG       AA      CU             C     C    \ --^    AG     A    -    AA-       G  AA
                                                120-270
>miR166w   Gm16:3661255:3661616:+
AAGTAGGGTGTTTGGAATGAAGTTTGATCCGAAATCATTGTCTCAGTTTCATTCATTTAATTTTAACTTTGCTTGTTGTCAGAGAGAGAGA
GATTTGTCATGATCTGAGAAGGAAAGGGGGTGTGTGGAATGAGGTTTGATCCAAGATCATCACATCATATATATATATATATATATATATA
TATATATATATATATATATATATATATATATATATATATATATATATATATCTTCTTCAATTTCCCTTTCCTTCTTGATTTAATTACTTACCT
TCATACATATACTGTTATTATTATCATCTCTTAATTCATGCATTGGATGGGACTGATCTCGGACCAGGCTTCATTCCTCACACCTAGCT
Initial ΔG = -130.50
A   A     UU             A      AAUCAUUGUC     UU     .-UUUAAUUUUAA     -|UU
 AGU GGGUGU  GGAAUGAAGUUUG UCCGA          UCAGU  CAUUCA                CUUUG C    \
 UCG UCCACA  CCUUACUUCGGAC AGGCU          AGUCA  GUAGGU                GAGAC G    G
-   A     CU             C      CU--------     GG     \ -----------         U^UU
                                                   90-310

> miR862c   Gm08:46853888:46854011:+
AACAGGAGAACTCTTCATGTTCCCTCAAAGGCTTCCAGTATTCCCGTATTCATTCATATATACCTAGCTAGAATTGATTGAA
TGCTGGATGTCTTTGAAGGAACTTGGAAGCTTACTTCGATCT
Initial ΔG = -42.00
AACA-    AACU    AU-     C         U          CCGU-|    AUUCAUAUA
     GGAG    CUUC   GUUCC UCAAAGGC UCCAGUAUUC          AUUC         \
     CUUC    GAAG   CAAGG AGUUUCUG AGGUCGUAAG          UAAG         U
UCUAG    AUUC    GUU     A         U          UUAGU^    AUCGAUCCA

> miR862d   Gm18:61624595:61624704:-
ATCAAAAGAAGTCTTCGTGTTCCCTCAAAGGCTTCCAGTATTCATTCATACCTAACTAGTTGCTTGAATGCTGGATGTCTTT
GAAGGAATTTGAAAGCTTACTTCTACGC
Initial ΔG = -42.50
AUCAA-   -|   UC    U     C         U           UU  UACCU
      AAG  AAG  UUCG GUUCC UCAAAGGC UCCAGUAUUCA CA      A
      UUC  UUC  AAGU UAAGG AGUUUCUG AGGUCGUAAGU GU      A
CGCAUC   A^   GA    U     A         U           UC  UGAUC
```

© 2013 by the authors; licensee MDPI, Basel, Switzerland. This article is an open access article distributed under the terms and conditions of the Creative Commons Attribution license (http://creativecommons.org/licenses/by/3.0/).
